# Phosphatidylinositol-phospholipase C3 negatively regulates the hypersensitive response via complex signaling with MAP kinase, phytohormones, and reactive oxygen species in *Nicotiana benthamiana*

**DOI:** 10.1093/jxb/erad184

**Published:** 2023-05-16

**Authors:** Shiori Takasato, Takuya Bando, Kouhei Ohnishi, Masayuki Tsuzuki, Yasufumi Hikichi, Akinori Kiba

**Affiliations:** Laboratory of Plant Pathology and Biotechnology, Faculty of Agriculture and Marine Science Kochi University, Nankoku, Kochi 783-8502, Japan; Laboratory of Plant Pathology and Biotechnology, Faculty of Agriculture and Marine Science Kochi University, Nankoku, Kochi 783-8502, Japan; Laboratory of Defense in Plant–Pathogen Interactions, Research Institute of Molecular Genetics, Kochi University, Nankoku, Kochi 783-8502, Japan; Laboratory of Plant Pathology and Biotechnology, Faculty of Agriculture and Marine Science Kochi University, Nankoku, Kochi 783-8502, Japan; Laboratory of Plant Pathology and Biotechnology, Faculty of Agriculture and Marine Science Kochi University, Nankoku, Kochi 783-8502, Japan; Laboratory of Plant Pathology and Biotechnology, Faculty of Agriculture and Marine Science Kochi University, Nankoku, Kochi 783-8502, Japan; University of Ghent, Belgium

**Keywords:** Hypersensitive response, jasmonic acid, MAP kinase, *Nicotiana benthamiana*, phosphatidylinositol-phospholipase C3, *Ralstonia solanacearum*, reactive oxygen species, salicylic acid, virus-induced gene silencing

## Abstract

Phospholipid signaling plays important roles in plant immune responses. Here, we focused on two phospholipase C3 (PLC3) orthologs in the *Nicotiana benthamiana* genome, *NbPLC3-1* and *NbPLC3-2*. We generated *NbPLC3-1* and *NbPLC3-2*-double-silenced plants (*NbPLC3s*-silenced plants). In *NbPLC3s*-silenced plants challenged with *Ralstonia solanacearum* 8107, induction of hypersensitive response (HR)-related cell death and bacterial population reduction was accelerated, and the expression level of *Nbhin1*, a HR marker gene, was enhanced. Furthermore, the expression levels of genes involved in salicylic acid and jasmonic acid signaling drastically increased, reactive oxygen species production was accelerated, and *NbMEK2*-induced HR-related cell death was also enhanced. Accelerated HR-related cell death was also observed by bacterial pathogens *Pseudomonas cichorii*, *P. syringae*, bacterial AvrA, oomycete INF1, and TMGMV-CP with L1 in *NbPLC3s*-silenced plants. Although HR-related cell death was accelerated, the bacterial population was not reduced in double *NbPLC3s* and *NbCoi1*-suppressed plants nor in *NbPLC3s*-silenced *NahG* plants. HR-related cell death acceleration and bacterial population reduction resulting from *NbPLC3s-*silencing were compromised by the concomitant suppression of either *NbPLC3*s and *NbrbohB* (respiratory oxidase homolog B) or *NbPLC3*s and *NbMEK2* (mitogen activated protein kinase kinase 2). Thus, NbPLC3s may negatively regulate both HR-related cell death and disease resistance through MAP kinase- and reactive oxygen species-dependent signaling. Disease resistance was also regulated by NbPLC3s through jasmonic acid- and salicylic acid-dependent pathways.

## Teaser

In *Nicotiana benthamiana*, PLC3-1 and -2 complement each other during the hypersensitive response to negatively regulate and fine tune the response through several signaling pathways, thereby preventing disruption of the immune response.

## Introduction

Plants have evolved sophisticated defense responses against phytopathogens, including preformed and active defenses (Thatcher *et al.,* 2004). Preformed defenses include physical barriers, such as thick cell walls and wax layers (Ziv *et al.* 2018), and a diverse array of secondary metabolites, many of which have antifungal activities. Some of these compounds are constitutive, existing in healthy plants in their biologically active forms. Others, such as cyanogenic glycosides and glucosinolates, occur as inactive precursors, and are activated in response to tissue damage or pathogen attack (Osbourn, 1996). In addition to preformed defenses, plants have a variety of active defense mechanisms to protect themselves from microbial pathogen infection. These responses include stomatal closure (Panchal and Melotto, 2017), reactive oxygen species (ROS) burst (Baker and Orlandi, 1995), cell wall glycoprotein cross-linking (Bradly *et al.*, 1992; Brisson *et al.*, 1994), lignification (Vance *et al.*, 1980), callose deposition (Voigt 2014), phytoalexin production (Ahuja *et al.* 2012; Jeandet 2015) and pathogenesis-related protein accumulation (Linthorst and van Loon, 2008). The most powerful defense response is the hypersensitive response (HR), which is accompanied by programmed cell death. HR is characterized by rapid, localized cell death triggered by an incompatible pathogen (Benoit and Dangl, 1997).

Plant defenses are initiated by the recognition of pathogen-derived molecules. A first example of pathogen recognition is the perception of pathogen-associated molecular patterns (PAMPs), leading to PAMP-triggered immunity (PTI). PAMPs have been recognized as generally conserved compounds, like chitin and glucan in fungi, and flagellins and lipopolysaccharides in bacteria. These PAMPs are recognized by plasma membrane-localized pattern-recognition receptors. PTI is induced by all invading pathogens (Bittel and Robatzek, 2007; Boller and He, 2009). The other mechanism of pathogen recognition is achieved through the recognition of pathogen effectors by resistance gene products (R proteins), followed by the induction of effector-triggered immunity (ETI; Jones and Dangl, 2006). PTI and ETI share signaling components that have distinct activation dynamics and amplitudes (Tsuda and Katagiri, 2010). Generally, PTI is characterized by broad-spectrum, transient and relatively mild immune responses without programmed cell death associated with the HR (Segonzac *et al.*, 2011; Bigeard *et al.*, 2014). In contrast, ETI is characterized by specific, sustainable, and robust immune responses with the HR (Tsuda and Katagiri, 2010).

After pathogen recognition, intracellular signal transduction cascades and transcriptional reprogramming are triggered during PTI and ETI. A ROS burst occurs rapidly through the respiratory burst oxidase homolog (RBOH), an NADPH oxidase that produces membrane-impermeable O_2_^.–^, and superoxide dismutase (SOD), which converts O_2_^.–^ into H_2_O_2_ in the apoplast (Li *et al.*, 2014; Podgórska *et al.*, 2017). H_2_O_2_ enters the cytosol and induces an elevation of cytosolic Ca^2+^ (Yuan *et al.*, 2017). Intracellular Ca^2+^ activates Ca^2+^-dependent protein kinases during the immune response in *Nicotiana benthamiana* and *N. tabacum* (Romeis *et al.*, 2001). Ca^2+^ activates H^+^/K^+^ ion fluxes, which lead to extracellular alkalinization and the depolarization of the plasma membrane (Jeworutzki *et al.*, 2010). Mitogen-activated protein kinase (MAPK) activation also occurs during immune signaling. MAPK activation leads to the phosphorylation of several transcription factors that regulate genes involved in ethylene, salicylic acid (SA) and jasmonic acid (JA) signaling, as well as antimicrobial compound production (Zhang and Klessig, 1998; Dahan *et al.*, 2009; Nishad *et al.*, 2020). This complex signaling network results in the establishment of PTI and/or ETI.

Phospholipid-based signaling cascades are another important component of intracellular signal transduction in plant immune responses. Phosphatidylinositol-specific phospholipase C (PI-PLC), a major component of phospholipid turnover, is an important lipid-hydrolyzing enzyme in both plants and animals. In plants, PLCs hydrolyze a specific substrate, phosphatidylinositol 4,5-bisphosphate, at the glycerophosphate ester linkages of membrane phospholipids, which leads to the generation of secondary messengers, such as diacylglycerol and inositol 1,4,5-trisphosphate (Singh *et al.*, 2015). We previously screened for PI-PLC orthologs in the completed *N. benthamiana* genome sequence (https://solgenomics.net/), and identified 12 PI-PLCs (Kiba *et al.*, 2020). Our objective is to clarify the roles of all 12 PLC orthologs in plant immunity. Our previous report showed that two NbPLC2 orthologs have important roles in pre- and post-invasion defenses, namely in PTI induction. During PTI induction, NbPLC2s can activate JA-mediated immune responses in plants, leading to the suppression of bacterial infections (Kiba *et al.*, 2020). In contrast, one of the NbPLC1 orthologs, NbPLC1-2, might have important roles in suppression of defense responses in HR, via negative regulation of JA- and ROS-mediated signaling (Ueta *et al.,* 2021). Thus, we hypothesized that there is role sharing between each PLC family member in plant defense regulation. In the present study, we focused on two PI-PLC3 orthologs, *NbPLC3-1* and *NbPLC3-2*, from the remaining 10 PI-PLCs. We determined the effects of silencing *NbPLC3-1*, *NbPLC3-2*, individually and together, on immune responses in *N. benthamiana*. We also discuss the regulatory roles of NbPLC3s on immune responses in *N. benthamiana*.

## Materials and methods

### Biological and chemical materials

*Nicotiana benthamiana* and *N. benthamiana NahG* plants were cultivated in a growth room as described previously (Maimbo *et al.*, 2007, 2010). *Ralstonia solanacearum* 8107 (Rs8107), *Pseudomonas cichorii*, and *P. syringae* pv. *syringae* were cultured in peptone yeast extract medium as described previously (Kiba *et al.*, 2003). *Agrobacterium tumefaciens* was cultured in YEB medium (Maimbo *et al.*, 2007). The bacterial populations of Rs8107, *P. cichorii*, and *P. syringae* pv. *syringae* were adjusted to 10^8^ colony forming unit (CFU) ml^–1^. Primers and plasmids used in this study are shown in Supplementary Tables S1 and S2, respectively.

### RNA isolation and cDNA synthesis

Total RNA was isolated from *N. benthamiana* leaves using a NucleoSpin RNA Plant kit (Macherey-Nagel, Düren, Germany). A 1-µg sample of total RNA was used as the template for reverse transcription using a PromeScript II 1^st^ strand cDNA Synthesis kit (TaKaRa Bio Co., Ltd., Shiga, Japan), as described by Maimbo *et al.,* (2007).

### Isolation of full-length cDNA of *NbPLC3-1 and NbPLC3-2*

PCR amplification was performed with the primer combination PLC3-1S and PLC3-1A for *NbPLC3-1*, and the combination PLC3-2S and PLC3-2A for *NbPLC3-2* (Supplementary Table S1), with 1 μg of cDNA from *N. benthamiana*. Cycling parameters were as follows: 30 cycles of 94 °C for 1 min, 55 °C for 2 min and 72 °C for 1 min. The full-length cDNAs were cloned into the pMD20 vector (TaKaRa Bio. Co., Ltd.) to generate pMD-NbPLC3-1 and pMD-NbPLC3-2.

### Sequencing

Sequence analysis was performed using M4 and RV primers (Supplementary Table S1) with the reagents for a Big Dye Terminator Cycle Sequencing Kit (Applied Biosystems, Foster, CA, USA) and an Applied Biosystems 3100 Avant Automated Sequencer (Applied Biosystems, Warrington, UK), in accordance with the manufacturer’s instructions. The sequence analysis was carried out using DNASIS (version 3.6; Hitachi, Yokohama, Japan) and the BLAST network service from the National Center for Biotechnology Information (Altschul *et al.*, 1990).

### Virus-induced gene silencing

Virus-induced gene silencing (VIGS) was performed as described previously (Mainbo *et al.*, 2007). The plasmids used for VIGS experiments are listed in Supplementary Table S2. cDNA sequences used for VIGS experiments were selected using the SGN VIGS tool (Fernandez-Pozo *et al*., 2014, 2015). cDNA fragments for *NbPLC3-1*, *NbPLC3-2*, and the combined *NbPLC3-1* + *NbPLC3-2* sequences (*NbPLC3*s) were amplified with the primers listed in Supplementary Table S1 using *N. benthamiana* cDNA as the template (Supplementary Fig. S1). These cDNA fragments were independently sub-cloned into the TA cloning site of pMD20 (TaKaRa Bio. Co., Ltd.) to generate pMD-NbPLC3-1, pMD-NbPLC3-2 and pMDNbPLC3s. These plasmids were digested with *Sal*I (TaKaRa Bio. Co., Ltd.) and ligated independently into *Sal*I-digested pPVX201 (Baulcombe *et al*., 1995). VIGS was conducted with pPVX201 harboring *NbPLC3-1* (pPVXPLC3-1), *NbPLC3-2* (pPVXPLC3-2), or *NbPLC3s* (pPVXPLC3s), independently. The pPVX201 plasmid lacking any inserts was used as a control as described previously (Maimbo *et al.,* 2007). These binary plasmids were transformed into *A. tumefaciens* strain GV3101, and inoculated into *N. benthamiana* and *N. benthamiana NahG* leaves. Three weeks after the initial *A. tumefaciens* inoculation, inoculations with *A. tumefaciens* carrying bacterial effector AvrA, oomycete elicitin, INF1, and coat protein from TMGMV with cognate L1 resistance protein, were performed on *N. benthamiana* leaves located three to four leaves above the *Agrobacterium*-inoculated leaf. The silencing efficiency levels were assessed using quantitative real-time PCR (qRT–PCR) assays (Kiba *et al.*, 2020).

### Bacterial population

The bacterial suspensions of *R. solanacearum* and *A. tumefaciens* were inoculated into *N. benthamiana* leaves using needleless syringes by the method described by Kiba *et al*. (2012). The bacterial populations were determined by plating on Hara–Ono plates (Maimbo *et al.*, 2010).

### Quantitative real-time PCR

Gene expression analysis was performed using qRT–PCR. Briefly, qRT–PCR was performed in 20 µl of reaction mixture, containing 1 µl of cDNA template, 10 pM of the respective primers (Supplementary Table S2) and THUNDERBIRD qPCR MIX (Toyobo Co. Osaka, Japan), on an Applied Biosystems (Thermo Fisher Scientific Inc., Tokyo, Japan) 7300 real-time PCR instrument. The cycling parameters were the same for all the primers: an initial 50 °C for 2 min and 95 °C for 10 min, followed by 40 cycles of 95 °C for 10 s and 60 °C for 1 min. Melting curve runs were performed at the end of each reaction to verify the specificity of primers, by detecting the presence of a single amplification product. The relative quantification of gene expression was performed in accordance with the instructions for the Applied Biosystems 7300 real-time PCR system, using the comparative cycle threshold [Ct] method to calculate the Qty value. We used two genes for internal standards (*NbUbe35* and *NbNQO*). When a pair of reference genes were used (NbUbe35/NbNQO), the geometric Cq mean and efficiency average were employed, as described previously (Pombo *et al.,* 2018).

### *Agrobacterium*-mediated transient expression

For agroinfiltration experiments, we used the binary vectors p35S-INF1, p35S-MEK2^DD^, p35S-AvrA, and p35S-TMGMV-CP, with p35S-L1 (Gupta *et al.,* 2013). These binary plasmids were transformed independently into *A. tumefaciens* strain GV3101. The resulting strains were inoculated into *N. benthamiana* leaves as described previously (Maimbo *et al.,* 2007).

### Estimation of cell death

Cell death was determined by measuring electrolyte leakage by ion conductivity (Gupta *et al.*, 2013) using a Twin Cord B-173 conductivity meter (HORIBA, Kyoto, Japan).

### ROS measurements using 3,3-diaminobenzidine staining

To determine the production of H_2_O_2_, 3,3’-diaminobenzidine (DAB) staining was performed as described by Gupta *et al.* (2013). *N. benthamiana* leaves infected with bacteria were infiltrated with 1 mg ml^−1^ of DAB solution (pH 3.8) and incubated for 1 h in the dark at 25 °C. The leaf samples were boiled in a solution of ethanol: acetic acid: glycerol (3:1:1). The leaf samples were then decolorized in 2.5 g ml^−1^ chloral hydrate solution for at least 30 min until the chlorophyll were completely removed.

### Immunoblot analysis

Leaf (~0.1 g) samples were homogenized in 200 μl SDS-PAGE sample buffer [50 mM Tris pH 7.9, 100 mM KCl, 1 mM EDTA, 20% (v/v) glycerol, and 1 mM DTT]. After centrifugation at 21 000 × *g* for 5 min, the clarified solution was used for protein analysis. Following separation of proteins using 10% SDS-PAGE, the appearance of phosphorylated MAPKs in the leaf extract was detected by western blotting using Phospho-p44/42 MAPK (Erk1/2) (Thr202/Tyr204) (197G2) rabbit monoclonal antibody (Cell Signaling Technology, MA, USA) according to the method described by Lee *et al.,* (2018). The large subunit of ribulose-1,5-bisphosphate carboxylase in samples, shown in the Ponceau S-stained gel, was used to monitor equal loading of protein.

### Statistical analysis

The statistical analysis was performed using *t*-tests (two-sided tests).

## Results

### Identification and characterization of PLC3s from *N. benthamiana*

On the basis of a phylogenetic analysis of the amino acid sequences of the PLCs, two PLC orthologs (Niben101Scf02280g02004.1 and Niben101Scf04093g00004.1) were classified into the same clade as PLC3 from *Solanum lycopersicum* (Kiba *et al.* 2020). Consequently, we designated them as *NbPLC3-1* (Niben101Scf02280g02004.1) and *NbPLC3-2* (Niben101Scf04093g00004.1). The deduced amino acid sequences of the full-length cDNAs of *NbPLC3-1* and *NbPLC3-2* contained complete PI-PLC-X, PI-PLC-Y and PI3K-C2 domains, which are required for enzymatic function of PLC3 from *Solanum lycopersicum* (Supplementary Fig. S2A, B).

### Effects of *NbPLC3-1* and *NbPLC3-2* silencing on the hypersensitive response towards *R. solanacearum*

To investigate the roles of *NbPLC3-1* and *NbPLC3-2* in plant immunity, VIGS of these genes was performed. We generated constructs for silencing *NbPLC3-1* (PLC3-1-VIGS), *NbPLC3-2* (PLC3-2-VIGS) and both *PLC3-1* and *PLC3-2* (PLC3s-VIGS). We estimated the suppression levels of *NbPLC3-1* and *NbPLC3-2* using qRT–PCR. Both *NbPLC3-1* and *NbPLC3-2* expression levels were reduced in the plants inoculated with *Agrobacterium* carrying the NbPLC3s-VIGS construct. In addition, the specific suppression of *NbPLC3-1* and *NbPLC3-2* expression was observed in the plants inoculated with *Agrobacterium* carrying NbPLC3-1-VIGS and NbPLC3-2-VIGS constructs, respectively (Supplementary Fig. S3A). In contrast, we could not observe significant suppression of expression of *NbPLC1-2* (Ueta *et al.* 2021), *NbPLC2-1*, and *NbPLC2-2* (Kiba *et al.,* 2020) closely related to *NbPLC3s* (Supplementary Fig. S3B).

**Fig. 1.**
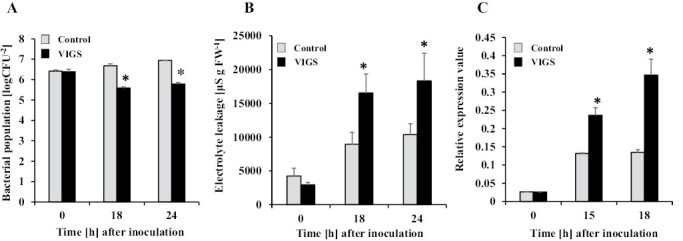
Responses of *NbPLC3s-*silenced plants to *Ralstonia solanacearum*. Empty vector control and *NbPLC3s-*silenced plant leaves were infiltrated with *R. solanacearum* strain 8107. (A) The bacterial populations of *R. solanacearum* 8107 in control (ray bar) and *NbPLC3s-*silenced (VIGS; black bar) plants were determined by plating at 18 and 24 h. Values are means ±SD of five replicate experiments. (B) Cell death induced by *R. solanacearum* was determined by measuring electrolyte leakage. Values are means ± SD (*n*=5). (C) Total RNA was isolated from *R. solanacearum* 8107-inoculated plants, and the expression levels of *Nbhin1* transcripts relative to the absolute non-treated control were normalized to internal standard genes (NbUbe35/NbNQO). Values represent means ±SD from triplicate experiments. Asterisks denote values significantly different from those of control plants (*; *P*<0.05, *t*-test).

We analyzed the function of *NbPLC3-1* and *NbPLC3-2* in immune responses using an *N. benthamiana–R. solanacearum* interaction model. We used Rs8107, which is an incompatible pathogen that induces the HR in *N. benthamiana*. The bacterial population was significantly reduced (*P*<0.05) in the *NbPLC3s*-silenced plants 18 h and 24 h after inoculation, compared with empty vector control plants (Fig. 1A). Induced cell death appeared in the control plants 18 h and 24 h after inoculation, whereas cell death acceleration was observed in *NbPLC3s*-silenced plants (Fig. 1B). The expression of the HR marker gene *Nbhin1* was also up-regulated in *NbPLC3*s plants (Fig. 1C). Thus, NbPLC3s might play important roles in the negative regulation of HR against Rs8107. In contrast, no significant changes in cell death induction or bacterial population were observed in individual *NbPLC3-1*- or *NbPLC3-2*-silenced plants (Supplementary Fig. S4A, B). Therefore, we used *NbPLC3s*-silenced plants for the functional analysis of NbPLC3s in the suppression of HR. We estimated HR-related cell death induction using *P. cichorii*, *P. syringae* pv. *syringae*, the *Agrobacterium*-mediated transient expression of *R. solanacearum* effector AvrA, the oomycete elicitin INF1, and TMGMV-CP with L1 (Huitema *et al*., 2005; Poueymiro *et al.*, 2009; Gupta *et al.* 2013). Similar to the HR induction caused by Rs8107, an acceleration of HR-related cell death was observed in *NbPLC3s-*silenced plants in response to inoculation with *P. cichorii*, *P. syringae*, as well as transient expression of AvrA, INF1 and TMGMV-CP with L1 (Supplementary Fig. S5).

### Silencing of *NbPLC3*s activates jasmonic acid and salicylic acid-dependent signaling in response to *R. solanacearum*

**Fig. 2.**
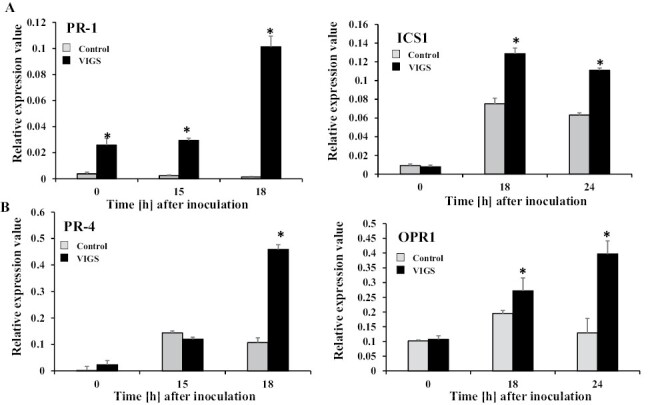
Up-regulation of jasmonic acid- and salicylic acid signaling pathways during accelerated HR in *NbPLC3s*-silenced plants. Empty vector control and *NbPLC3s-*silenced plant leaves were infiltrated with *R. solanacearum* strain 8107. Total RNA was isolated from control and *NbPLC3s*-silenced plants at 0, 15, 18, or 24 h following inoculation with *R. solanacearum* 8107. Expression values of (A) salicylic acid-related *PR-1* and *ICS1*, and (B) jasmonic acid-related *PR-4* and *OPR1* are shown as relative expression after normalization to internal standard genes (NbUbe35/NbNQO). Values represent means ±SD from triplicate experiments. Asterisks denote values significantly different from those of control plants (*; *P*<0.05, *t*-test).

We previously reported that the HR in response to *R. solanacearum* is at least partially regulated by JA and SA signaling (Kiba *et al*., 2003; Gupta *et al.*, 2013). Thus, we investigated the influence of *NbPLC3* silencing on JA and SA signaling. Total RNA was extracted from *NbPLC3s*-silenced and control plants at 0, 18, and 24 h after inoculation with Rs8107. As shown in Fig. 2A, the expression level of *PR-1*, a marker gene for the SA signaling pathway, was significantly increased (*P*<0.05) in *NbPLC3s*-silenced plants, compared with empty vector control plants challenged with Rs8107. In addition, the expression of *NbICS1*, encoding the SA biosynthetic enzyme isochorismate synthase 1, was also up-regulated in *NbPLC3s*-silenced plants. Thus, NbPLC3s may be involved in the SA-mediated defense signaling cascade against *R. solanacearum*.

Next, we investigated the effects of *NbPLC3* silencing on JA signaling. The expression of *PR-4*, a marker gene for the JA signaling pathway, was significantly up-regulated (*P*<0.05) in *NbPLC3s*-silenced plants challenged with Rs8107, compared with control plants. The expression of *NbOPR1*, encoding the JA biosynthetic enzyme 12-oxophytodienoate reductase 1, was also increased in *NbPLC3s*-silenced plants (Fig. 2B). Thus, NbPLC3s may be involved in JA-mediated immune responses against *R. solanacearum*.

### Silencing of *NbPLC3s* activates SA-dependent disease resistance against *Ralstonia solanacearum*

**Fig. 3.**
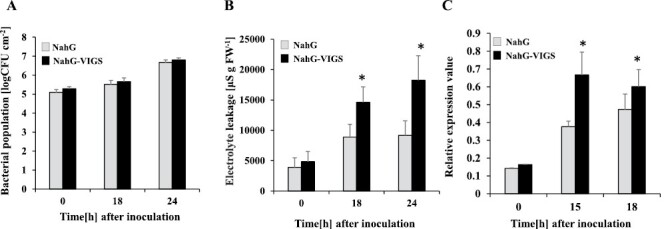
Role of the salicylic acid pathway in accelerated HR in *NbPLC3s*-silenced plants. *NahG* and *NbPLC3s*-silenced *NahG* (*NahG*-VIGS) plants were infiltrated with *R. solanacearum* strain 8107. (A) Bacterial populations were determined by plating at 0, 18, or 24 h following inoculation. Values are means ±SD of four replicate experiments. (B) *NahG* and *NahG*-VIGS plants were infiltrated with *R. solanacearum* 8107. Cell death induction was determined by measuring electrolyte leakage. Values are means ±SD of four replicate experiments. Asterisks denote values significantly different from those of empty vector control plants (C) Total RNA was isolated from NahG and NahG-VIGS plants at 0, 15, and 18 h after inoculation with *R. solanacearum* 8107. Expression values of *Nbhin1* are shown as relative expression after normalization to internal standard genes (NbUbe35/NbNQO). Values represent means ±SD from triplicate experiments. Asterisks denote values significantly different from those of empty vector control plants (*; *P*<0.05, *t*-test).

To further analyze the involvement of SA in the accelerated HR caused by silencing *NbPLC3s*, we used plants expressing a SA-degrading enzyme (*NahG* plants). The enhanced disease resistance phenotype was not observed in *NbPLC3s*-silenced *NahG* plants, because no significant changes in bacterial growth were observed in either *NahG* or *NbPLC3s-*silenced *NahG* plants (Fig. 3A). An acceleration in HR-related cell death in response to Rs8107 infiltration was observed in *NbPLC3s*-silenced *NahG* plants compared with empty vector control *NahG* plants (Fig. 3B). The expression of the HR marker *Nbhin1* was also up-regulated in *NbPLC3s-*silenced *NahG* plants compared with *NahG* control plants after inoculation with Rs8107 (Fig. 3C). Thus, NbPLC3s may regulate resistance induction through SA-mediated signaling against *R. solanacearum*. The acceleration of HR-related cell death by *NbPLC3s*-silencing may not be related to SA-dependent signaling, since *NahG* plants could not accumulate SA.

### Silencing of *NbPLC3s* activates JA-dependent defenses against *R. solanacearum*

**Fig. 4.**
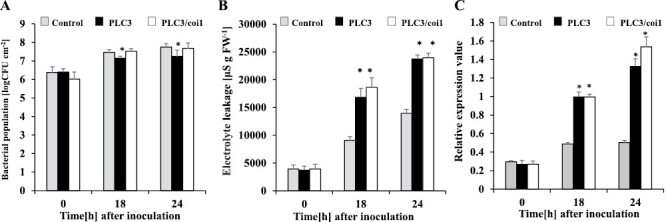
Role of the jasmonic acid pathway in accelerated HR in *NbPLC3s*-silenced plants. Empty vector control, *NbPLC3s* (PLC3s) and *NbPLC3s:NbCoi1* double-knockdown (PLC3/coi1) plant leaves were infiltrated with *R. solanacearum* strain 8107. (A) Bacterial populations were determined in control, PLC3s, and PLC3/coi1 plants by plating at 0, 18, and 24 h following inoculation. Values are means ±SD of four replicate experiments. Asterisks show significant differences (*P*<0.05) between the control, *NbPLC3*s and double-knockdown plants. (B) Cell death induction was determined by measuring the ion conductivity levels in control, PLC3s, and PLC3s/coi1 plants. Values are means ±SD of four replicate experiments. (C) Total RNA was isolated from control, PLC3s, and PLC3s/coi1 plants at 0, 18, and 24 h after inoculation with *R. solanacearum* 8107. Expression values of *Nbhin1* are shown as relative expression after normalization to internal standard genes (NbUbe35/NbNQO). Values represent means ±SD from triplicate experiments. Asterisks denote values significantly different from those of control plants (*; *P*<0.05, *t*-test).

Because *NbPLC3s*-silenced plants showed the rapid activation of JA-dependent *PR-4* expression, as well as JA biosynthetic *NbOPR1* expression in response to Rs8107 infection, we further analyzed the roles of the JA pathway in accelerated HR caused by silencing *NbPLC3*. We generated silenced plants for *NbPLC3s* as well as *NbCoi1* (*NbPLC3s*:*Coi1*-silenced plants), which encodes an F-box protein required for JA signaling (Zhai *et al*., 2015). The enhanced disease resistance phenotype was weaker in *NbPLC3s*:*Coi1*-silenced plants compared with *NbPLC3s*-silenced plants, because there was more bacterial growth in *NbPLC3s*:*Coi1*-silenced plants than in *NbPLC3s*-silenced plants, and the bacterial population recovered to that seen in empty vector control plants (Fig. 4A). An acceleration in HR-related cell death in response to Rs8107 infiltration was observed in *NbPLC3s*:*Coi1*-silenced plants, similar to that seen in *NbPLC3s*-silenced plants (Fig. 4B). The expression of *Nbhin1* also increased in both *NbPLC3s*- and *NbPLC3s*:*Coi1*-silenced plants (Fig. 4C). There was no change in bacterial population and cell death induction when only *NbCoi1* was silenced, compared with control plants (Supplementary Fig. S6). Although the acceleration of HR-related cell death by *NbPLC3s*-silencing may not be related to JA-dependent signaling, the enhanced disease resistance may be regulated through JA-mediated signaling against *R. solanacearum* in *NbPLC3s*-silenced plants (Fig. 4).

### Silencing of *NbPLC3*s activates reactive oxygen species burst in response to *R. solanacearum*

**Fig. 5.**
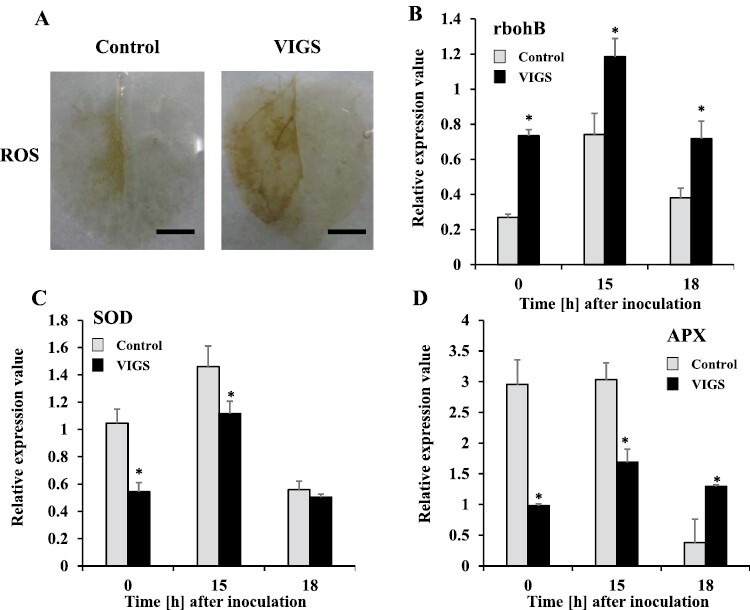
Hyper-production of reactive oxygen species during accelerated HR in *NbPLC3s*-silenced plants. Empty vector control and *NbPLC3s-*silenced plant leaves were infiltrated with *R. solanacearum* strain 8107. (A) ROS production visualized 18 h after inoculation as assessed by DAB staining. These experiments were repeated with 10 biological replicates, and representative results are shown. Scale bars=1 cm. (B–D) Total RNA was isolated from control and *NbPLC3s*-silenced plants at 0, 15, and 18 h after inoculation with *R. solanacearum* 8107. Expression values of *rbohB*, *APX1* and *SOD* are shown as relative expression after normalization to internal standard genes (NbUbe35/NbNQO). Values represent means ±SD from triplicate experiments. Asterisks denote values significantly different from those of control plants (*; *P*<0.05, *t*-test).

Plants undergoing HR usually produce ROS (Dumnovic *et al*., 2021). Therefore, we evaluated ROS production in control and *NbPLC3s*-silenced plants in response to Rs8107 infection. The staining of H_2_O_2_ with DAB revealed more brown patches in *NbPLC3s*-silenced plants, which were comparable to those on control plants infected with Rs8107. Increased DAB staining was observed in *NbPLC3s*-silenced plants in response to Rs8107 infection (Fig. 5A). ROS levels are regulated by the balance between ROS production and elimination (Dumnovic *et al*., 2021). Subsequently, we analyzed the expression pattern of a gene encoding a major ROS-producing enzyme, NbrbohB, which is a membrane-bound NADPH oxidase (Yoshioka *et al.,* 2003). *NbrbohB* expression level increased at 15 h after the inoculation of control plants with Rs8107, and significantly increased (*P*<0.05) in *NbPLC3s*-silenced plants challenged with Rs8107, compared with empty vector control plants (Fig. 5B). Next, we investigated the effects of *NbPLC3s-*silencing on the expression levels of genes encoding the ROS scavenging enzymes ascorbate peroxidase (*NbAPX*) and superoxide dismutase (*NbSOD*). Although the expression of *NbSOD* was up-regulated in both control and *NbPLC3s*-silenced plants at 15 h after inoculation with Rs8107, a significant reduction (*P*<0.05) in *NbSOD* expression was observed in *NbPLC3s*-silenced plants compared with empty vector control plants (Fig. 5C). In addition, the expression level of *NbAPX* was significantly reduced (*P*<0.05) in *NbPLC3s*-silenced plants 0–15 h after inoculation with Rs8107, compared with control plants (Fig. 5D). Thus, NbPLC3s may be involved in the ROS-mediated defense signaling cascade against *R. solanacearum*. In addition, the hyper-accumulation of ROS in *NbPLC3s*-silenced plants may result from both the induced expression of a ROS biosynthetic enzyme, and reduced expression of ROS scavenging enzymes.

### Silencing of *NbPLC3*s activates ROS-dependent defense signaling in response to *R. solanacearum*

**Fig. 6.**
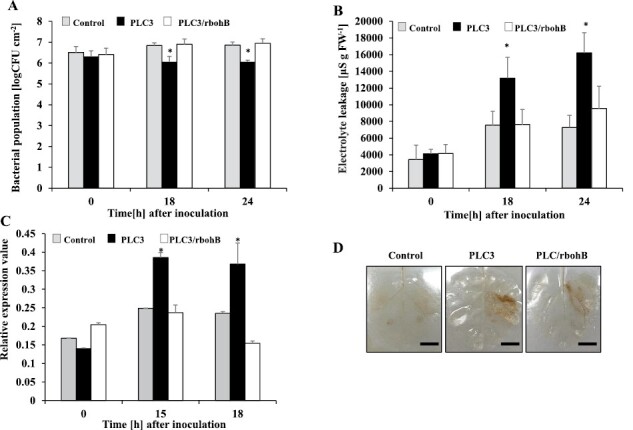
Role of reactive oxygen species in accelerated HR in *NbPLC3s*-silenced plants. Empty vector control, *NbPLC3*s (PLC3), and *NbPLC3s:NbrbohB* double-knockdown (PLC3/rbohB) plant leaves were infiltrated with *R. solanacearum* 8107. (A) Bacterial populations were determined in control, PLC3, and PLC3/rbohB plants by plating at 0, 18, and 24 h following inoculation. Values are means ±SD of four replicate experiments. Asterisks show significant differences among control, PLC3, and PLC3/rbohB plants (*P*<0.05, *t*-test). (B) Cell death induction was determined at 0, 18, and 24 h by measuring the ion conductivity levels in the control, PLC3s, and PLC3s/rbohB plants. Values are means ±SD of four replicate experiments. Asterisks show significant differences among control, PLC3, and PLC3/rbohB plants (*P*<0.05, *t*-test). (C) Total RNA was isolated from control, *NbPLC3s* (PLC3), and *NbPLC3s:NbrbohB* double-knockdown (PLC3/rbohB) plants at 0, 15, and 18 h after inoculation with *R. solanacearum* 8107. Expression values of *Nbhin1* are shown as relative expression after normalization to internal standard genes (NbUbe35/NbNQO). Values represent means ±SD from triplicate experiments. Asterisks denote values significantly different from those of control plants (*; *P*<0.05, *t*-test). (D) ROS production was seen 18 h after inoculation as assessed by DAB staining. These experiments were repeated with 10 biological replicates, and representative results are shown. Scale bars=1 cm.

Increased ROS accumulation and elevated levels of *NbrbohB* expression were observed in *NbPLC3s*-silenced plants (Fig. 5A, B). To determine whether the increased HR was caused by NbrbohB-dependent accumulation of ROS, we generated plants in which both *NbPLC3s* and *NbrbohB* were silenced (*NbPLC3s*:*rbohB* plants). The enhanced disease resistance phenotype was weaker in *NbPLC3s*:*rbohB*-silenced plants compared with *NbPLC3s*-silenced plants, because the bacterial population recovered to that seen in empty vector control plants (Fig. 6A). There was no change in bacterial population and cell death induction in only *NbrbohB*-silenced plants when compared with control plants (Supplementary Fig. S6). Intriguingly, the HR-related cell death phenotype, as well as enhanced *Nbhin1* expression, in response to Rs8107 infiltration, were also compromised in *NbPLC3s*:*rbohB* plants (Fig. 6B, C). In *NbPLC3s*:*rbohB* plants, elevated ROS production was compromised in response to Rs8107 infection, and ROS accumulation returned to approximately that seen in empty vector control plants (Fig. 6D). These results indicate that elevated ROS production through NbrbohB is involved in both the acceleration of HR-related cell death induction and enhanced disease resistance in *NbPLC3s*-silenced plants.

### Silencing of *NbPLC3s* activates MAPK-dependent defense signaling in response to *R. solanacearum*

**Fig. 7.**
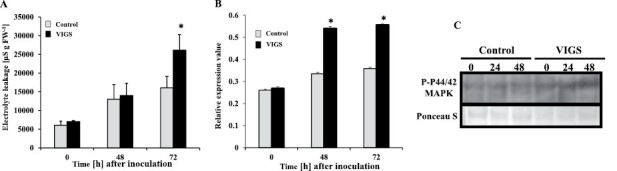
Relation of the MAPK pathway in accelerated HR cell death in *NbPLC3s*-silenced plants. Empty vector control and *NbPLC3s-*silenced plant leaves were infiltrated with *Agrobacterium tumefaciens* harboring 35S-MEK2^DD^. (A) Cell death induction was determined by measuring the ion conductivity levels in control and *NbPLC3s-*silenced (PLC3s) plants. Values are means ±SD of four replicate experiments. (B) Total RNA was isolated from control and PLC3s plants at 0, 48, and 72 h after inoculation with *A. tumefaciens* harboring 35S-MEK2^DD^. Expression values of *Nbhin1* are shown as relative expression after normalization to internal standard genes (NbUbe35/NbNQO). Values represent means ± SD from triplicate experiments. Asterisks denote values significantly different from those of control plants (*; *P*<0.05, *t*-test). (C) MAPK activation in control and VIGS plants was analyzed by immunoblots with anti-P-p44/42 MAPK (T202/Y204) antibodies at 0, 24, and 48 h after inoculation. Protein loading was monitored by Ponceau S staining of the bands corresponding to ribulose-1,5-bisphosphate carboxylase large subunit.

**Fig. 8.**
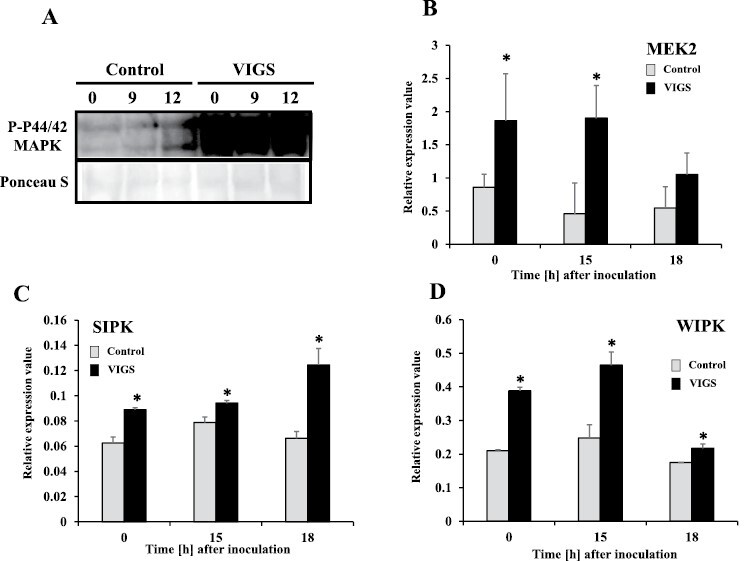
Hyper-induction of MAP kinase cascade during the accelerated HR in *NbPLC3s*-silenced plants. Empty vector control and *NbPLC3s-*silenced plant leaves were infiltrated with *R. solanacearum* strain 8107. (A) MAPK activation was analyzed by immunoblots with anti-P-p44/42 MAPK (T202/Y204) antibody 0, 9, and 12 h after inoculation. (B–D) Total RNA was isolated from control and *NbPLC3s*-silenced plants at 0, 15, and 18 h after inoculation with *R. solanacearum* 8107. Expression values of *MEK2*, *SIPK* and *WIPK* are shown as relative expression after normalization to internal standard genes (NbUbe35/NbNQO). Values represent means ±SD from triplicate experiments. Asterisks denote values significantly different from those of control plants (*; *P*<0.05, *t*-test).

MAPK cascades are sufficient to induce the HR in *N. benthamiana* (Yoshioka *et al.*, 2009). To evaluate the roles of the MAPK cascade in accelerating the HR in *NbPLC3s*-silenced plants, we used *Agrobacterium* carrying a constitutively active form of MAPK kinase 2 (*NbMEK2*^*DD*^; Katou *et al.*, 2003). The induction of HR-related cell death was observed in both control and *NbPLC3s*-silenced plants at 48 h and 72 h after inoculation. A strong induction of HR-related cell death was observed in *NbPLC3s*-silenced plants at 72 h after inoculation with *A. tumefaciens* expressing *NbMEK2*^*DD*^, compared with control plants (Fig. 7A). The induction of *Nbhin1* expression was significantly accelerated (*P*<0.05) in *NbPLC3s*-silenced plants in comparison with control plants at 48 h and 72 h after inoculation (Fig. 7B). To investigate how *NbPLC3s*-silencing affected the MAPK cascade, we analyzed activation of MAPK by immunoblot analysis. We observed phosphorylated MAPK as immunoreactive bands similar in size to SA-induced protein kinase (SIPK: 48 kDa) and wound-inducible protein kinase (WIPK: 46 kDa) by *Agrobacterium*-mediated expression of *NbMEK2*^*DD*^. Phosphorylated MAPKs were slightly increased even at the 0 h time point in *NbPLC3s*-silenced plants in comparison with control plants, and increased at 24 h and 48 h after expression of *NbMEK2*^*DD*^ (Fig. 7C). Phosphorylated MAPKs were also observed in both control and *NbPLC3s*-silenced plants in response to Rs1807 inoculation. Increased phosphorylated MAPKs were observed at 9 h and 12 h after inoculation with Rs8107 in *NbPLC3s*-silenced plants in comparison with control plants (Fig. 8A). Expression analysis of *NbMEK2* showed that enhanced expression was observed at 0 h and 15 h after inoculation with Rs8107 in *NbPLC3s*-silenced plants in comparison with control plants (Fig. 8B). The expression of SIPK was enhanced at 0 h and 18 h after inoculation with Rs8107 (Fig. 8C). Transcriptional activation of WIPK was also observed at 0 h and 18 h after inoculation with Rs8107 (Fig. 8D). Therefore, MAPK signaling cascade appears to be enhanced by *NbPLC3s*-silencing.

**Fig. 9.**
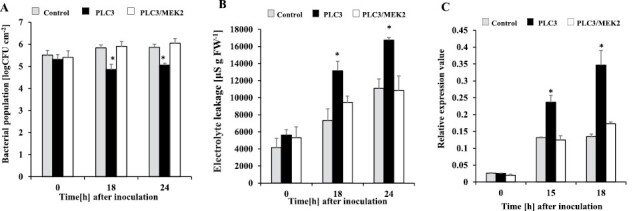
Role of the MAPK pathway in accelerated HR in *NbPLC3s*-silenced plants. Empty vector control, *NbPLC3s-*silenced (PLC3s), and *NbPLC3s:NbMEK2* double-knockdown (PLC3/MEK2) plant leaves were infiltrated with *R. solanacearum* strain 8107. (A) Bacterial populations were determined in control, PLC3s, and PLC3/MEK2 plants by plating at 0, 18, and 24 h after inoculation. Values are means ±SD of four replicate experiments. Asterisks indicate significant differences among control, PLC3s, and PLC3/MEK2 plants (*P*<0.05, *t*-test). (B) Cell death induction was determined by measuring the ion conductivity levels in control, PLC3s, and PLC3s/MEK2 plants. Values are means ±SD of four replicate experiments. (D) Total RNA was isolated from control, PLC3s, and PLC3/MEK2 plants at 0, 15, and 18 h after inoculation with *R. solanacearum* 8107. Expression values of *Nbhin1* are shown as relative expression after normalization to internal standard genes (NbUbe35/NbNQO). Values represent means ±SD from triplicate experiments. Asterisks denote values significantly different from those of control plants (*; *P*<0.05, *t*-test).

To determine whether the acceleration of the HR by Rs8107 in *NbPLC3s*-silenced plants involved the MAPK cascade, we generated plants in which both *NbPLC3s* and *NbMEK2* were silenced (*NbPLC3s:MEK2* plants). The enhanced disease resistance phenotype was compromised in *NbPLC3s*:*MEK2* plants compared with *NbPLC3s*-silenced plants, because the bacterial population recovered to numbers seen in control plants (Fig. 9A). Both the acceleration of HR-related cell death and *Nbhin1* expression in response to Rs8107 were compromised in the *NbPLC3s:MEK2* plants (Fig. 9B, C). We observed no significant change in bacterial population and cell death induction in single *NbMEK2*-silenced plants when compared with control plants (Supplementary Fig. S6). Therefore, the MAPK cascade might be involved in both acceleration of HR-related cell death induction and disease resistance in *NbPLC3s*-silenced plants.

## Discussion

Plant PI-PLCs have been implicated in a number of cellular processes and signal transduction events (Pokotylo *et al.*, 2014). Nine PI-PLCs, PI-PLC1–9, have been identified in *Arabidopsis thaliana* (Tasma *et al.* 2008). Recently, PI*-*PLCs have been identified in the tomato genome and classified into seven groups (Abd-El-Haliem *et al.*, 2016). We previously found 12 PI-PLC orthologs, classified into seven groups, in *N. benthamiana* (Kiba *et al.*, 2020). In this research, we analyzed two PI-PLC3 orthologs, *NbPLC3-1* (Niben101Scf02280g02004.1) and *NbPLC3-2* (Niben101Scf04093g00004.1; Supplementary Fig. S2A). The deduced amino acid sequences of the full-length cDNAs of *NbPLC3-1* and *NbPLC3-2* contained complete PI-PLC-X, PI-PLC-Y, and PI3K-C2 domains, which are required for enzymatic activity, suggesting that both *NbPLC3-1* and *NbPLC3-2* encode an enzymatically active form of PI-PLC3 (Supplementary Fig. S2B, C). PLCs convert phosphatidylinositol 4,5-biphosphate (PIP2) into inositol 1,4,5-triphosphate (IP3) and diacylglycerol (DAG; Don *et al*., 2012). DAG is rapidly converted to phosphatidic acid (PA) in plant cells. PA might act as an intracellular second messenger to activate downstream signaling enzymes such as RboH and PDK (Putta *et al.,* 2016). PA is a key lipid signaling molecule, and is involved in stress responses, in metabolism, and in development (Testerlink *et al*., 2011). Another PLC product, IP3, participates in the response to various abiotic stresses, gravitropism, phototropism, and auxin transport (Salinas-Mondragon *et al*., 2010; Zhang *et al*., 2011). Further analysis is required to understand enzymatic function, substrates, products, and downstream signaling related to NbPLC3s.

PI-PLCs affect biotic stress responses, including plant immunity (Canonne *et al.,* 2011). Both the flagellin-triggered response and the internalization of the corresponding receptor, FLS2, of *A. thaliana*, are suppressed by the inhibition of PI-PLC activities (Abd-El-Haliem *et al.,* 2016). *PI-PLC2*-silenced Arabidopsis plants are susceptible to the type III secretion system-deficient bacterial strain of *Pseudomonas syringae* pv. *tomato* DC3000 (*hrc*C-), indicating the role of PI-PLC2 in stomatal pre-invasive defenses (D’Ambrosio *et al.,* 2017). Our previous report also showed that PI-PLC2 orthologs, NbPLC2s, may have important roles in pre- and post-invasive defenses, namely in the induction of PTI in *N. benthamiana* (Kiba *et al.,* 2020). Thus, PLCs may play important roles in PTI induction. Several plant PI-PLC families are required for HR-mediated immune responses, including ETI (Canonne *et al.*, 2011). Treatment with fungal xylanase leads to the rapid induction of PI-PLC activity and consequent HR in tomato suspension cells, and induction of HR is required to increase *SlPLC5* expression (Raho *et al.*, 2011). SlPLC2 is reportedly required for the HR because HR-related *Hsr203J* expression is suppressed in *SlPLC2*-silenced tomato plants (Gonorazky *et al.*, 2014). The silencing of *SlPLC6* results in HR reduction and increased colonization by *Cladosporium fulvum* in Cf-4 tomato plants. Avr4-induced HR is also compromised in *SlPLC4-*silenced Cf-4-carrying tomato. In addition, the heterologous expression of *SlPLC4* results in an accelerated Avr4/Cf-4-induced HR in *N. benthamiana* (Abd-El-Haliem *et al.*, 2016). Thus, HR and/or ETI induction are dependent on the functions of PI-PLC members. Intriguingly, our data showed that the silencing of *NbPLC3*s accelerated HR induction after inoculation with bacterial pathogens Rs8107 and *P. cichorii*, *P. syringae* pv. *phaseolicola*, and the transient expression of the bacterial effector AvrA and oomycete effector INF1 (Fig. 1; Supplementary Fig. S5). Accelerated HR was also observed by the concomitant expression of TMGMV-CP with L1 (Fig. 1; Supplementary Fig. S5). Thus, NbPLC3s may play important roles in the negative regulation of HR and/or ETI. Intriguingly, no changes in HR induction were observed in *NbPLC3-1-* or *NbPLC3-2-*individually silenced plants (Supplementary Fig. S4). Therefore, NbPLC3-1 and NbPLC3-2 may cooperatively regulate HR and/or induction. It is also possible that the activity of just one NbPLC3 is not sufficient to suppress the HR. In addition, the expression of *NbPLC3-1* and *NbPLC3-2* in *NbPLC3-2*-silenced and *NbPLC3-1*-silenced leaves, respectively, was significantly higher than the controls (Supplementary Fig. S3A). Therefore, NbPLC3-1 and NbPLC3-2 may mutually complement each other at the transcriptional level during the development of HR.

In plants, JA and SA are required for defense responses against *R. solanacearum*. Extracellular polysaccharides from *R. solanacearum* trigger the expression of genes involved in SA-mediated defenses in resistant tomato plants (Milling *et al.*, 2011). The RipB effector strongly induces the production of SA-related defensive genes and the HR in *N. benthamiana* (Nakano and Mukaihara, 2019). The RipE1 effector induces the expression of JA-responsive genes and JA biosynthesis (Sang *et al.*, 2020). Our previous report also showed an induction of both SA- and JA-mediated responses in response to Rs8107 in *N. tabacum* (Kiba *et al.*, 2003, 2014). Therefore, drastic changes in the JA and SA signaling pathways may be required for immune responses against *R. solanacearum*. In fact, the overexpression of *NtWRKY50* enhances bacterial resistance, which correlates with enhanced SA and JA signaling-related gene expression levels (Liu *et al.*, 2017). A JA-dependent signaling pathway is required for *Pythium oligandrum*-induced resistance against *R. solanacearum* in tomato (Hase *et al.*, 2008). SA induces disease resistance against *R. solanacearum* in tomato as a result of cell wall strengthening, through the deposition of lignin and the induction of defense-related enzymes (Mandal *et al.*, 2013). The SA signaling pathway also plays a significant role in defense against *R. solanacearum* in several *Solanum* lines (Baichoo and Jaufeerally-Fakim, 2017). In this study, we showed an enhancement in disease resistance accompanied by the up-regulation of both JA and SA signaling in *NbPLC3s-*silenced plants (Figs 2–4). Therefore, NbPLC3s may negatively regulate disease resistance through these plant hormone signaling pathways during the HR against *R. solanacearum*. Inversely, cell death acceleration may not be regulated through JA and SA signaling by NbPLC3s (Figs 3, 4). These results provide novel insights into the regulatory roles of PI-PLC3s in phytohormone signaling during the HR.

ROS generation has been implicated in HR induction (Gechev and Hille 2005; Asai and Yoshioka, 2009; Yoshioka *et al.*, 2009). In plants, NADPH oxidases, designated as RBOHs, have been identified as being encoded by genes related to mammalian gp91^phox^ in various plants (Groom *et al.*, 1996; Torres *et al.*, 2002; Yoshioka *et al.*, 2003). The silencing of *NbrbohB* compromises ROS generation during the HR, and NbrbohB-dependent ROS production has a significant role in HR induction (Yoshioka *et al.*, 2003). In contrast, aerobic organisms have evolved antioxidant defenses that include catalases, peroxidases, and SODs, to minimize the damaging effects of ROS (Dumanovic *et al.*, 2021). Plant tissue antioxidants counterbalance the damaging effects of ROS (Dey *et al.*, 2020). However, if a severe infection occurs and the antioxidants fail to scavenge the over-produced ROS, then cell death occurs. Consequently, an elevation in the antioxidant potential of a plant should enhance its tolerance to the development of cell death caused by pathogens (Barna *et al.*, 1993). In the present study, the acceleration of HR may have been due to the activation of NbrbohB-dependent ROS production, because HR-related cell death acceleration and enhanced disease resistance were compromised in the *NbPLC3s* and *NbrbohB* VIGS lines (Figs 5,6). In addition, reductions in *NbAPX* and *NbSOD* expression were also observed in *NbPLC3s*-silenced plants, indicating that the suppression of antioxidant enzymes might be involved in ROS hyper-production, leading to the accelerated HR in *NbPLC3s*-silenced plants (Fig. 5). Therefore, NbPLC3s may have dual regulatory roles in ROS generation, by affecting both ROS-generating and ROS scavenging systems during the HR. These results provide novel insights into the regulatory roles of PLC3 in the counterbalancing of ROS during the HR.

In addition to ROS signaling, members of the MAPK family, SIPK, WIPK, and NTF6 are involved in HR induction in response to PAMPs, INF1, and hyphal wall components from *Phytophthora infestans* in *N. benthamiana* (Yoshioka *et al.*, 2003; Asai *et al.*, 2008). Both WIPK and SIPK are also required to induce the *N*-gene-mediated HR to tobacco mosaic virus (Jin *et al.*, 2003). LeMKK2, LeMKK3, and LeMPK3 are required for Pto-mediated HR against *P. syringae* pv. *tomato* carrying AvrPto (Ekengren *et al.*, 2003). Our present results showed that HR-related cell death induction by *NbMEK2*^*DD*^ expression was accelerated significantly in the *NbPLC3s*-silenced plants (Fig. 7). Activation of WIPK and SIPK was also enhanced in the *NbPLC3s*-silenced plants. In addition, transcriptional activation of *MEK2*, *SIPK*, and *WIPK* was observed in the *NbPLC3s*-silenced plants (Figs 7, 8). Furthermore, co-silencing of *NbPLC3s* and *NbMEK2* compromised both accelerated HR-related cell death and bacterial population reduction in *NbPLC3s*-silenced plants inoculated with Rs8107 (Fig. 9). Therefore, the MAPK cascade might also participate in accelerated HR through *NbPLC3s*-silencing, and could be up-regulated in *NbPLC3s*-silenced plants at the transcriptional and post-translational level. Further analyses of the relationship between PLC3 and the MAPK cascade need to be performed to clarify the complex signaling interaction between phospholipids and MAPKs.

Plants have evolved sophisticated immune regulatory systems controlled by complex mechanisms (Tsuda and Katagiri, 2010). Based on the results with *coi1*, *rbohB*, *MEK2*-silenced plants, and *NahG* plants, we also suggest that these signaling components might regulate immune responses in a complex manner, and cannot drastically affect HR induction during the Rs8107 and *N. benthamiana* interaction by a single signaling cascade (Supplementary Fig. S6). The immune regulatory switching mechanisms might turn on immune responses after pathogen attacks. The switch follows with a deactivating ‘off’ signal to avoid self-inflicted damage to the plant (Lu *et al.,* 2018; Mengarelli *et al.*, 2021). Our previous report showed that other PLC orthologs, NbPLC2s, contribute to the induction of pre- and post-invasive PTI responses in plants (Kiba *et al.*, 2020). Conversely, our present findings suggest that NbPLC3s negatively regulate plant immune responses, by suppression of HR. Therefore, the PLC family might switch the immune responses ‘on’ and ‘off’ during pathogen attack, which allows a cost-efficient way to prevent undesirable immune responses. Further studies are necessary to clarify the complex NbPLC-mediated signaling networks involved in switching plant immune signaling cascades ‘on’ and ‘off’.

From our findings, we conclude that while undergoing HR induction, NbPLC3s may be suppressing JA- and SA-mediated signaling as part of disease resistance suppression. In addition, NbPLC3s may down-regulate both HR-related cell death and disease resistance through the MAPK cascade and ROS production pathway in *N. benthamiana.* Further studies are necessary to clarify the complex NbPLC3-mediated signaling networks that act through MAPK, ROS, JA, and SA to characterize the phospholipid turnover involved in the HR signaling cascade. In summary, NbPLC3s act as negative HR regulators and fine tune the HR to prevent immune disruption, which has undesirable developmental effects.

## Supplementary data

The following supplementary data are available at *JXB* online.

Fig. S1. Nucleotide sequences of *NbPLC3-1* and *NbPLC3-2.*

Fig. S2. Characterization of phosphatidylinositol-specific phospholipase C3 (PI-PLC3) in *Nicotiana benthamiana*.

Fig. S3. Estimation of expression value of *NbPLC3-1*, *NbPLC3-2*, *NbPLC1-2*, *NbPLC2-1*, NbPLC2-2, *Nbcoi1*, *NbrbohB,* and *NbMEK2*.

Fig. S4. Effects of *NbPLC3-1-* and *NbPLC3-2*-specific silencing on bacterial population and hypersensitive cell death induction by incompatible *Ralstonia solanacearum* 8107.

Fig. S5. Effects of *NbPLC3s*-silencing on HR induction by *Pseudomonas cichorii*, *Pseudomonas syringae* pv. *phaseolicola*, AvrA, INF1, and TMGMV-CP with L1.

Fig. S6. Bacterial population and hypersensitive cell death induction by incompatible *Ralstonia solanacearum* 8107 in *Nbcoi1*, *NbrbohB* and *NbMEK2*-silenced plants and *NahG* plants.

Table S1. Primers used in this study.

Table S2. List of plasmids used in this study.

erad184_suppl_Supplementary_Figures_S1-S7_Tables_S1-S2

## Data Availability

All data supporting the findings of this study are available within the paper and within its supplementary data published online.

## Abbreviations

CFU: colony forming unit
DAB: 3,3’-diaminobenzidine
ETI: effector-triggered immunity
HR: hypersensitive response
JA: jasmonic acid
MAPK: mitogen-activated protein kinase
PA: phosphatidic acid
PAMPs: pathogen-associated molecular patterns
PLC: phospholipase C
PTI: pathogen-associated molecular pattern-triggered immunity; ROS; reactive oxygen species
SA: salicylic acid
VIGS: virus-induced gene silencing.
