# Supplementary material for: Phosphatidylinositol-phospholipase C3 negatively regulates the hypersensitive response via complex signaling with MAP kinase, phytohormones, and reactive oxygen species in Nicotiana benthamiana
Source: J Exp Bot. 2023 May 16;74(15):4721–35. doi: 10.1093/jxb/erad184 (PMC10433933; doi:10.1093/jxb/erad184)
Supplement: erad184_suppl_Supplementary_Figures_S1-S7_Tables_S1-S2 [file erad184_suppl_supplementary_figures_s1-s7_tables_s1-s2.pdf]

Fig. S1

```
NbPLC3-1 (Niben101Scf02280) ATTCTTTTATACGCGGAATAACCACCAAAACTTTTATACCTTCCCATCTTCCTAAGCCAAAAA--------------AACCGTTACTTCTA-ACAATCACATAAATCATTTCTCCTCTC 104
NbPLC3-2 (Niben101Scf04093) ---CAACAATCCATT--ATAATCCC---AATTTTTATACCTTGCCATTTTCCTAAGCAAAAAACAAAAAAGAAGAAAAAACCGTTATCTCTTCATAATCACATAAATCATTTCTCCTCTC 112

NbPLC3-1 (Niben101Scf02280) CTCAAAAAATCATCCTCCTCCGTTTTCCGTTTTCC-------TCTTTCAAACAATGTCGAGACAGACGTACAGAGTCTGTTTCTGTTTCCGGCGGCGGTTCCGGGTAGTCGCCGCCGAGG 217
NbPLC3-2 (Niben101Scf04093) CTCAACAAATCATCCCTCTCCGTTTTCCGTTTTCCGTTTTCCTCTTTCAAACAATGTCGAGACAGACGTACAGAGTCTGTTTCTGTTTCCGGCGGCGGTTCCGAGTAGTCGCCGCCGAGG 232

NbPLC3-1 (Niben101Scf02280) CTCCAGCGGATGTAAAGAATTTATTCAATAGATACTCCGATAACGGAGTAATGAATGCAGAGAACCTACAACGATTCTTAATTGAGGTTCAGAAAGAGGAAAATGCGAGTTTAGAGGATG 337
NbPLC3-2 (Niben101Scf04093) CTCCGGCGGATGTAAAGAATTTATTCAATAGATATTCCGATAACGGAGTAATGAATGCAGAGAACCTACAATGATTCTTAATTGAGGTTGAGAAAGAGGAAAATGCGAGTTTAGAGGATG 352

NbPLC3-1 (Niben101Scf02280) CTCATGGTATTATG---------------AAT-AATCTTCATG-ACCT------------------------------------TAAGATCCTTAA---TATTTTTCATCGGAGAGGTT 400
NbPLC3-2 (Niben101Scf04093) CTCAGGGTATTTCGTTCGTCGCCTTCTGCAGTGAATCTTGCTGCAGCTGCAGCTGCAGCTGCATTGGTAGTACGCCAAGTTGAAACAAAATACTCTGCTCTGCTTTTCAA-GCAGCAACT 471

                                                                                                                   PLC3s-VIGS
NbPLC3-1 (Niben101Scf02280) TACATC--------------TTGACGCATTTTTTAAGTAT--CTTTTTGCTGATATTA--ATC-CTCCTATCAATCCTAAACGCGGGATTCACCATGATATGAATGAGCCTTTGTCTCAT 501
NbPLC3-2 (Niben101Scf04093) TACAGCATATGTTGAAAAGATTTATGAAATTATTAGGGATAACTTGAAGAAGGAGTTGGGATCACTCCTTTC---CTTATGCTCCAGATTCACCATGATATGAATGAGCCTTTGTCTCAT 588

NbPLC3-1 (Niben101Scf02280) TACTTCATATACACAGGACATAATTCTTATCTAACTGGGAATCAACTAAGTAGTGATTGCAGTGATGTTCCCATAATTCAAGCCCTGCACCGAGGTGTACGAGTAATTGAATTGGATATA 621
NbPLC3-2 (Niben101Scf04093) TACTTCATATACACAGGACATAATTCTTATCTAACCGGGAATCAACTGAGTAGTGATTGCAGTGATGTCCCCATAATACAAGCCCTGCACCAAGGTGTACGAGTAATTGAATTGGATATA 708

NbPLC3-1 (Niben101Scf02280) TGGCCAAATTCCGCCAAAGATGATGTGGAAGTTTTGCATGGACGAACATTGACCACTCCAGTTGCGCTCATCAAATGTCTAAAGTCTATCAAGGAACATGCTTTTTCTGTATCTGAATAT 741
NbPLC3-2 (Niben101Scf04093) TGGCCAAATTCCGCCAAAGATTACGTGGAAGTTTTGCATGGAGGAACATTGACCACTCCGGTTGCGCTCATCAAATGTCTGAGGTCTATAAAGGAACATGCTTTTACTGTATCAGAGTAT 828

NbPLC3-1 (Niben101Scf02280) CCTGTTGTAATAACACTTGAAGATCATCTAACTCAAGATCTTCAGGCAAAAGTTGCGGAGATGATCACTCAAACATTTGGAGACATGCTGTTTTCTCCCGATTCTTGTTTGAAGGATTTC 861
NbPLC3-2 (Niben101Scf04093) CCTGTTGTGGTAACACTTGAAGATCATTTAACCCCAGATCTTCAGGCAAAAGTTGCAGAGATGATCACTCAAACATTTGGAGACATGCTGTTTTCTCCCGATTCATGTTTGAAAGACTTT 948

NbPLC3-1 (Niben101Scf02280) CCCTCCCCGGAATCACTGAAAAGACGCGTTTTGATATCAACTAAGCCGCCCAAAGAATATCTTCAGGCGAAGGAAGTTAAGGAAAAAGACTCGAAGAAAGGAACGGAGTCACCTGATACA 981
NbPLC3-2 (Niben101Scf04093) CCCTCCCCAGAATCTCTGAAAAGACGTGTTCTGATATCAACTAAGCCACCCAAAGAATACCTTCAAGCGAAGGAAGTTAAGGAAAAAGACTCGAAAAAAGGAACGGATTCACCTGATTCA 1068

NbPLC3-1 (Niben101Scf02280) GAAGCTTGGGGAAGAGAAGTTTCAGACCTTAAAGCCAGATACAATGATAAG---GATGATTCTGATGATGGAGTAGGTGATGAAGATGAATTAAGCGATGAAGGAGATCCCAATTCACAG 1098
NbPLC3-2 (Niben101Scf04093) GAAGCTTGGGGAAGGGAAGTTTCAGACCTTAAAGCCAGATATAACGATAAGCAGGATGATTCTGATGAAGGAGCAGGCGAGGAAGATGATGGAAGTGATGAAGGAGATCCCAACTCGCAG 1188

NbPLC3-1 (Niben101Scf02280) CAAAATGTAGCACCAGAATACAAGCGTTTAATTGCCATTCATGCTGGAAAAGGAAAAGGTGGACTGTCAGATTGGCTGAGGGTTGATCCTGATAAAGTAAGACGGCTTAGCTTGAGTGAG 1218
NbPLC3-2 (Niben101Scf04093) CAAA-TGTCGCACCAGAATACA-----------------TATGCTGGAAAGGGAAAAGGTGGATTGTCAGATTGGCTGAGGGTTGATCCTGATAAAGTAAGACGACTTAGCTTGAGCGAG 1290

NbPLC3-1 (Niben101Scf02280) CAAGAACTTGGAAAGGCTGTAGTTACTCATGGAAAAGAAATTATCAGGTTCACTAAGAGGAACTTGCTGAGAATATACCCAAAGGGCATACGTTTTGACTCATCTAATTACAACCCTCTT 1338
NbPLC3-2 (Niben101Scf04093) CAAGAACTTGGAAAGGCTGTAGTTACTCATGGAAAAGAAATTATCAGGTTCACTCAGCGGAACTTGCTCAGAATATACCCAAAGGGCATACGTTTTGACTCATCCAATTAT---CCTTTT 1407

NbPLC3-1 (Niben101Scf02280) GTTGCATGGGCGCATGGAGCTCAAATGGTGGCATTCAATATGCAGGGCTATGGAAGATCACTTTGGTTAATGCATGGTATGTTCAGATCCAATGGTGGTTGTGGATATGTTAAGAAACCA 1458
NbPLC3-2 (Niben101Scf04093) GTTGCATGGACGCATGGAGCTCAAATGGTGGCATTCAATATGCAGGGCTATGGAAAATCACTTTGGTTAATGCATGGTATGTTCAGATCCAATGGTGGTTGTGGATATGTTAAGAAACCA 1527

NbPLC3-1 (Niben101Scf02280) GATATACTATTGAAAGCGGGTCCCAACAATCAGGTTTTCGATCCTGAAGCAAATTTGCCAGTCAAAACTACATTGAAGGTGACCGTATTTATGGGTGAAGGATGGTATTATGATTTCAAG 1578
NbPLC3-2 (Niben101Scf04093) GATATACTATTGAAAGCAGGTCCTAACAATCAGATC----ATCTT---------TTG--------------------------GTATTTA------------GCATTA--ATTTT--- 1589

NbPLC3-1 (Niben101Scf02280) CACACACACTTTGATGCATACTCGCCTCCAGATTTCTATGCAAAGATAGGAATTGCCGGAGTTCTGGCTGATAATATAATGAAGAAAACAAAGACTCTTGAGGACAATTGGATACCAACT 1698
NbPLC3-2 (Niben101Scf04093) --------CTTTAAAGTACATT----TCAAG--TTGGAAGTTAAGATAGGAATTGCCGGAGTTCCCGCAGATAATGTAATGAAGAAAACAAAGACACTGGAGGATAATTGGATACCAACT 1695

NbPLC3-1 (Niben101Scf02280) TGGGATGAAAAGTTTGAGTTCCCATTAACAGTTCCTGAGTTGGCTCTACTTCGCGTCGAAGTTCATGAGTATGATATGTCTGAAAAAGATGATTTTGCTGGCCAAACTTGTTTACCTGTT 1818
NbPLC3-2 (Niben101Scf04093) TGGGATGAAAAGTTTGAGTTCCCATTAACAGTTCCTGAATTGGCTCTACTTCGCGTCGAAATTCATGAGTATGATATGTCTGAAAAGGATGATTTTGCTGGCCAAACTTGTTTACCTGTT 1815

                                                                                                                         PLC3-1/3-2-VIGS
NbPLC3-1 (Niben101Scf02280) TCAGAACTAAGACAAGGCATCCGAGCAGTTTCACTCCACGACCGAAAGGGAGAGAAATACAACTCTGTGAAGCTTCTTATGCGCTTCGAATTTGTCTAACTGTGAGTCGCTTGTTTTCTC 1938
NbPLC3-2 (Niben101Scf04093) TCAGAACTAAGACAAGGTATTCGAGCAGTTTCACTCCACGACCGAAAGGGAGAGAAATA-AACTCTGTGAATCTTCTTATGCGTTTCGAATTTGTCTAACTGTGAGTCGTTTGTTTTCTC 1934

NbPLC3-1 (Niben101Scf02280) TTATGTTTCTGTGTTTGTTGTGGTAAATTCT-GTATCTTTGTGAGTATGTG-AGATGAGATGTAAATAAATATATGTAGTAAGATGTGTGCAAATATTTTTTGGAAAGTTGTCTGTCTCT 2056
NbPLC3-2 (Niben101Scf04093) TTATGTTCCTGTGTATGTTGTGGTAAATTTTTGTATCTTTGTCAATAAGTTTGGGTGAGATGTAAATAAATATATGTGGTAAAATGTGTGCAAATATTTTTTGGAAAGTCGTCTGTCTCT 2054

NbPLC3-1 (Niben101Scf02280) TTGTAACTTGTG------GTAAATTCTG-----TAAATAGATGTGTGTGGTAATATGTGTGAAATTAATTTTTGAAAATTCGTTCTCATTTTCATGGTAGTTTGTAATGCTGCAATCAAG 2165
NbPLC3-2 (Niben101Scf04093) TTGTAACTTCTGTAATACATAAGCTCTGAGATGTAAATAGATGTAT--GGTAATATGTGTGAAATTA---------------TGTCATTTCAGTGGTGGTTTATATTGCTGCAATCAAA 2156

NbPLC3-1 (Niben101Scf02280) TAAAACATATATGGGCGCTAATCATGGGATTATTTTTATTTATCTAAGCTTTAGGTGGACAAAATTATCTAATACTACCTGTTGTTGAACAATTCAGGTGCGTGCAAATCAATTTGAACA 2285
NbPLC3-2 (Niben101Scf04093) TAAAACATAGATAGGCGACAACAAGGAAA--------------CCAAGGTTCA---------AATTA-----TAGTATC--------AACAA--------------AAT-------GATA 2219

NbPLC3-1 (Niben101Scf02280) CTGCCG 2291
NbPLC3-2 (Niben101Scf04093) ------
```

Figure S1 Nucleotide sequences of *NbPLC1-1* and *NbPLC1-2*.
cDNA fragments used for VIGS experiments are shown with gray backgrounds.

Fig. S2

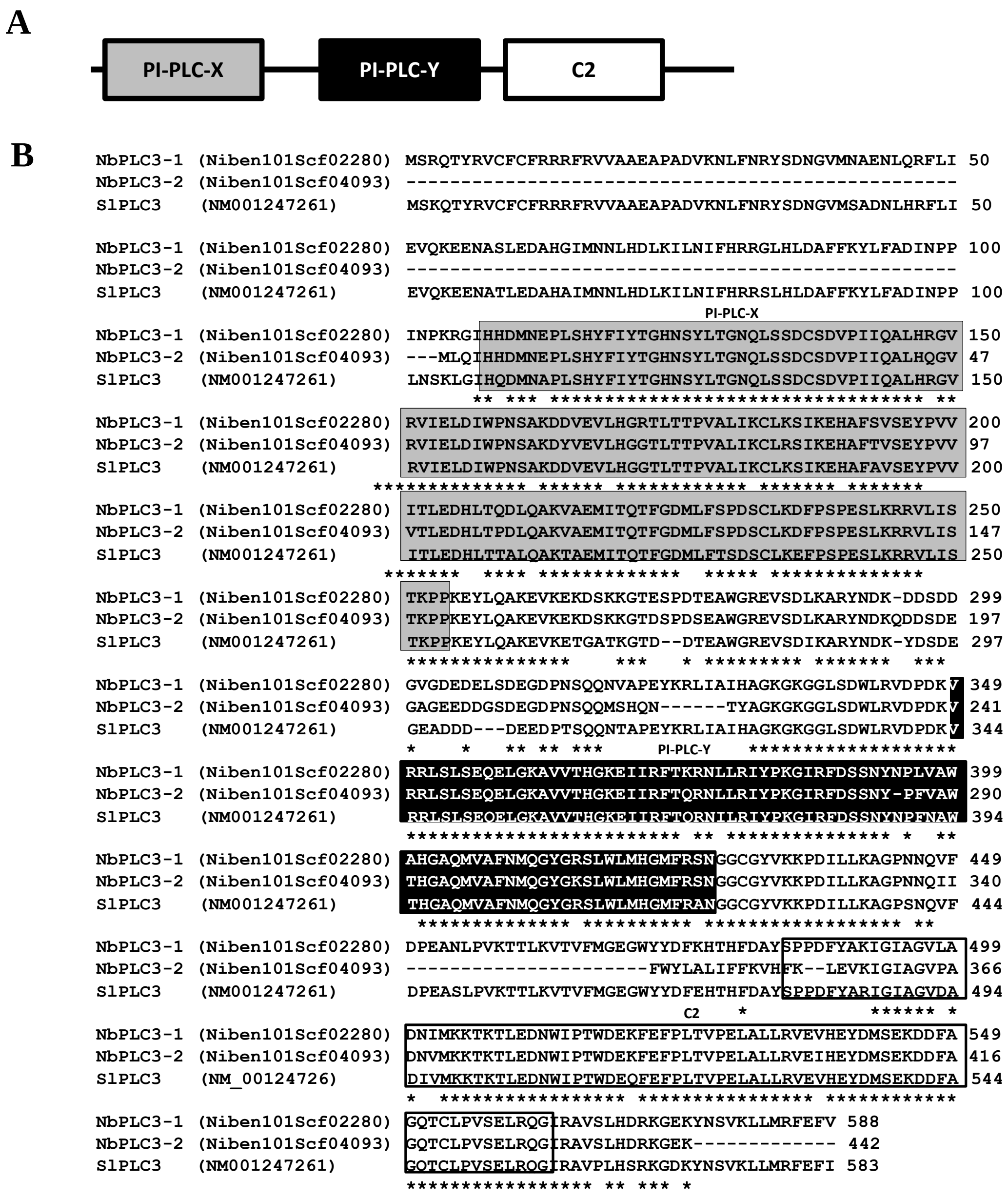

Figure S2 Characterization of phosphatidylinositol-specific phospholipase Cs (PI-PLCs) in *Nicotiana benthamiana*.

(A) Schematic representation of the PLC3 protein structure. PI-PLC-X, PI-PLC-Y and PI3K-C2 domains are the conserved domain essential for PLC activities. (B) Alignment of deduced amino acid sequences of *NbPLC3-1* (Niben101Scf02280g02004.1), *NbPLC3-2* (Niben101Scf04093g00004.1) and *Solanum lycopersicum* PLC3 (*SlPLC3,* NM001247261). Asterisks showed identical amino acids. Gray, black and white boxes indicate PI-PLC-X, PI-PLC-Y and PI3K-C2 domains, respectively.

Fig. S3

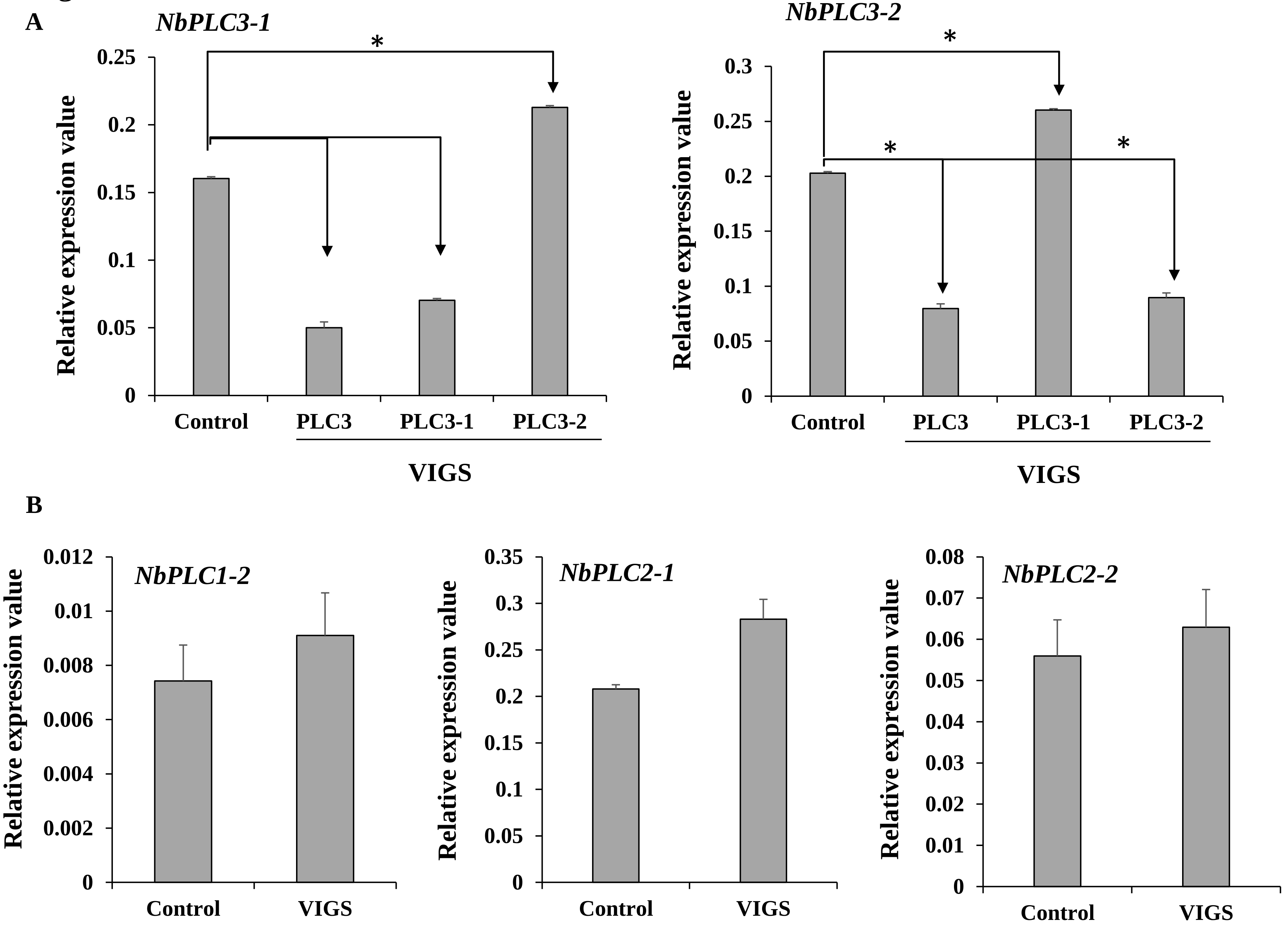
A
NbPLC3-1
Relative expression value
0.25
0.2
0.15
0.1
0.05
0
*
Control
PLC3
PLC3-1
PLC3-2
VIGS
NbPLC3-2
Relative expression value
0.3
0.25
0.2
0.15
0.1
0.05
0
*
*
*
Control
PLC3
PLC3-1
PLC3-2
VIGS
B
NbPLC1-2
Relative expression value
0.012
0.01
0.008
0.006
0.004
0.002
0
Control
VIGS
NbPLC2-1
Relative expression value
0.35
0.3
0.25
0.2
0.15
0.1
0.05
0
Control
VIGS
NbPLC2-2
Relative expression value
0.08
0.07
0.06
0.05
0.04
0.03
0.02
0.01
0
Control
VIGS

Fig. S3 continued

C

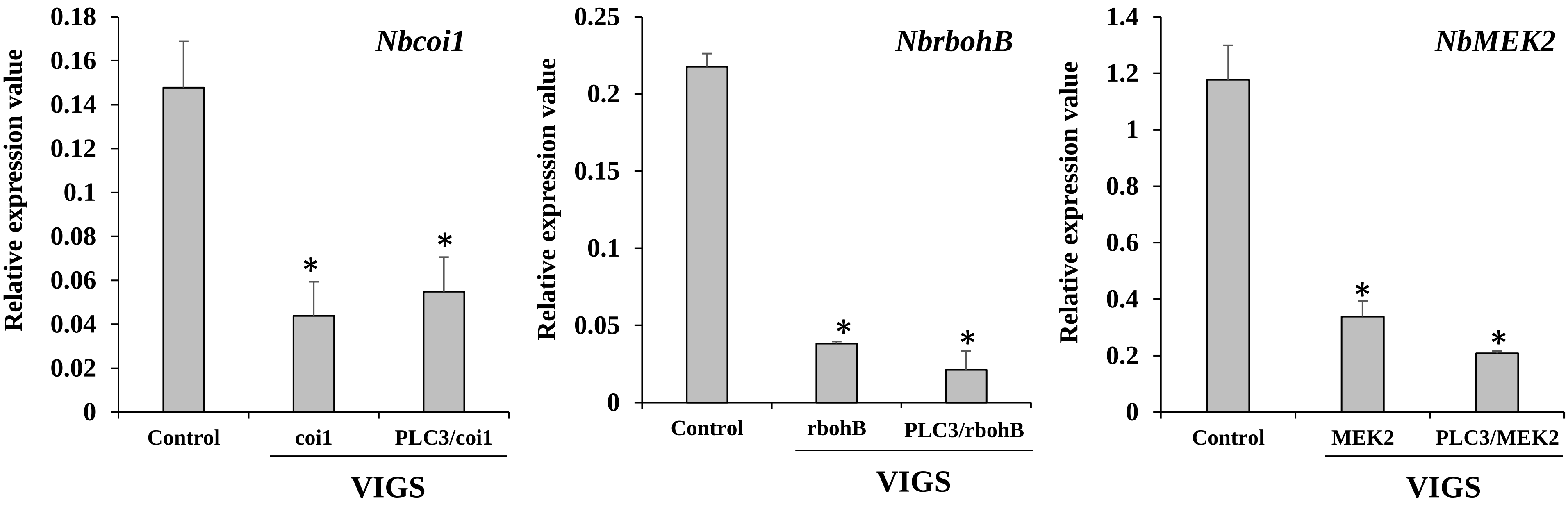

Estimation of expression value of *NbPLC3-1*, *NbPLC3-2*, *NbPLC1-2*, *NbPLC2-1*, NbPLC2-2, *Nbcoi1*, *NbrbohB* and *NbMEK2*.

(A) Total RNAs were isolated from empty vector control, *NbPLC3-1*- (VIGS:PLC3-1), *NbPLC3-2*- (VIGS:PLC3-2) and both *NbPLC3-1*- and *NbPLC3-3*- (VIGS:PLC3) silenced plants. Transcript levels of *NbPLC3-1* and *NbPLC3-2* were estimated by qRT-PCR. Values represent means ± SDs from triplicate experiments. (B) Total RNAs were isolated from empty vector control and both *NbPLC3-1*- and *NbPLC3-2*- (VIGS) silenced plants. Transcript levels of *NbPLC1-2*, *NbPLC2-1* and *NbPLC2-2* were estimated by qRT-PCR. (C) Total RNA was isolated from empty vector control (Control), *Nbcoi1* (coi1) and *NbPLC3s:Nbcoi1* double-knockdown (PLC3/coi1), *NbrbohB* (rbohB) and *NbPLC3s:NbrbohB* double-knockdown (PLC3/rbohB), *NbMEK2* (MEK2) and *NbPLC3s:NbMEK2* double-knockdown (PLC3/MEK2) plants. Transcript levels of *Nbcoi1*, *NbrbohB* and *NbMEK2* were estimated by qRT-PCR. Transcripts relative to the absolute non-treated control were normalized to internal standard genes (NbUbe35/NbNQO). Values represent means ± SDs from triplicate experiments.

Fig. S4

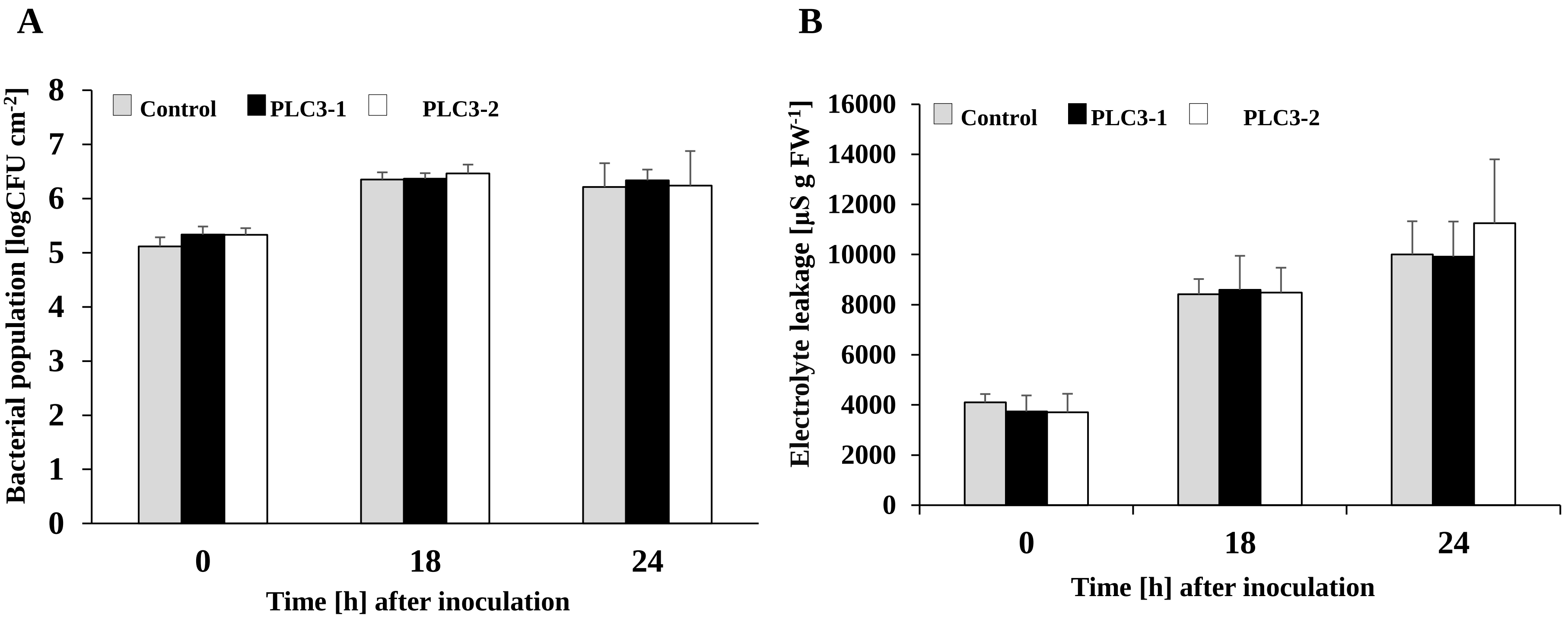

Figure S4 Effects of *NbPLC3-1*- and *NbPLC3-2*-specific silencing on hypersensitive response (HR) induction by incompatible *Ralstonia solanacearum* 8107.

Control, *NbPLC3-1*- and *NbPLC3-2*-silenced plant leaves were infiltrated with *R. solanacearum* strain 8107. (A) The bacterial populations of *R. solanacearum* 8107 in Control (gray bar), *NbPLC3-1*-silenced (VIGS; black bar) and *NbPLC3-2*-silenced (VIGS; white bar) plants were determined by plating at specified time points. Values are means ± SDs of five replicate experiments. (B) Cell death induced by *R. solanacearum* was determined in Control (gray bar), *NbPLC3-1*-silenced (VIGS; black bar) and *NbPLC3-2*-silenced (VIGS; white bar) plants by measuring the ion conductivity levels. Values represent means ± SDs (n = 5).

Fig. S5

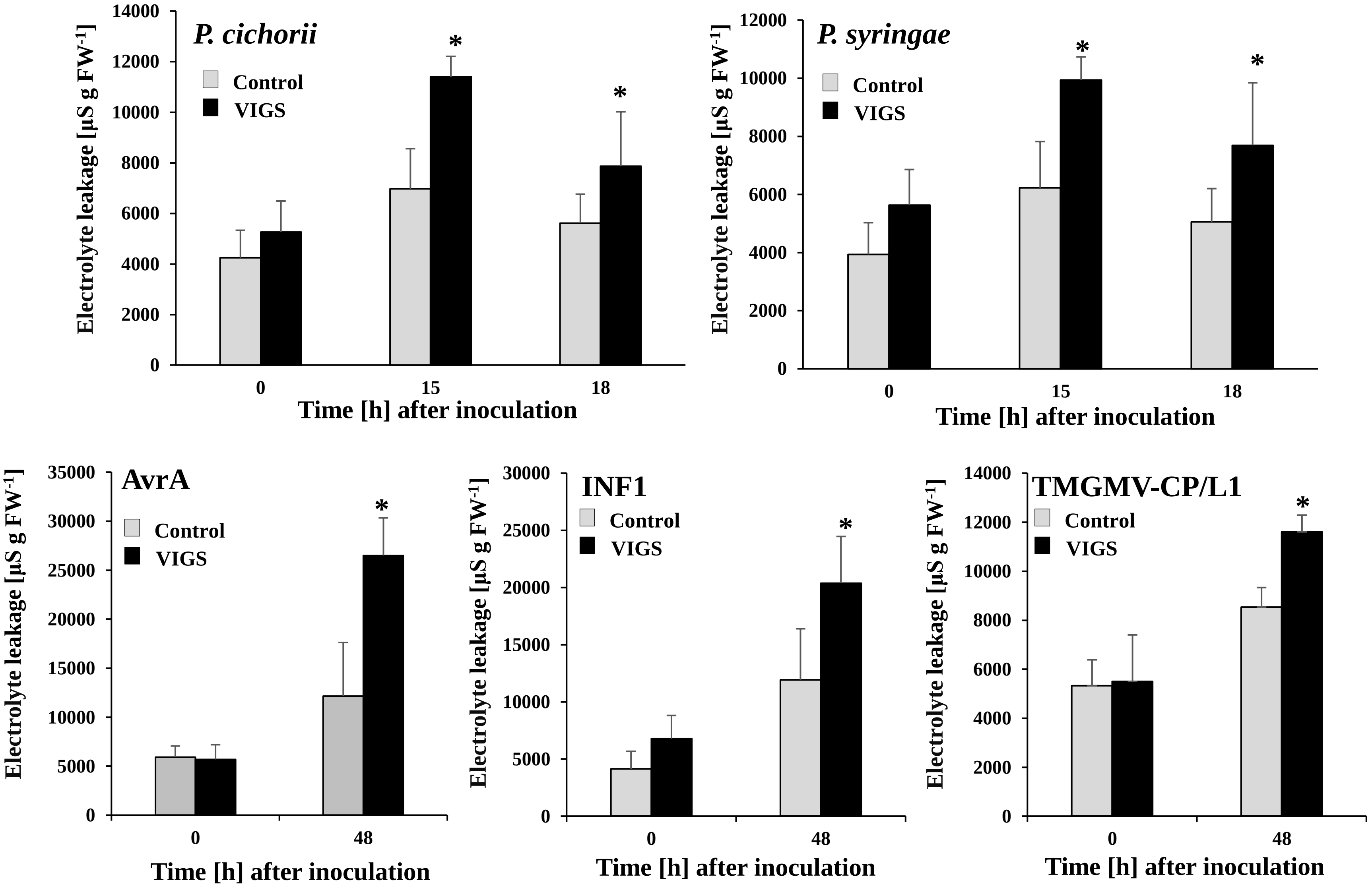

Figure S5. Effects of *NbPLC3s*-silencing on HR induction by *Pseudomonas cichorii*, *Pseudomonas syringae* pv. *phaseolicola*, AvrA, INF1 and TMGMV-CP with L1.

Control and *NbPLC3s*-silenced *N. benthamiana* plants were infiltrated with *Pseudomonas cichorii*, *P. syringae*, *Agrobacterium* harboring independently 35S-AvrA (AvrA) and 35S-INF1 (INF1). Cell death induction was determined by measuring the ion conductivity level. Values represent means ± SDs (n = 5).

Fig. S6

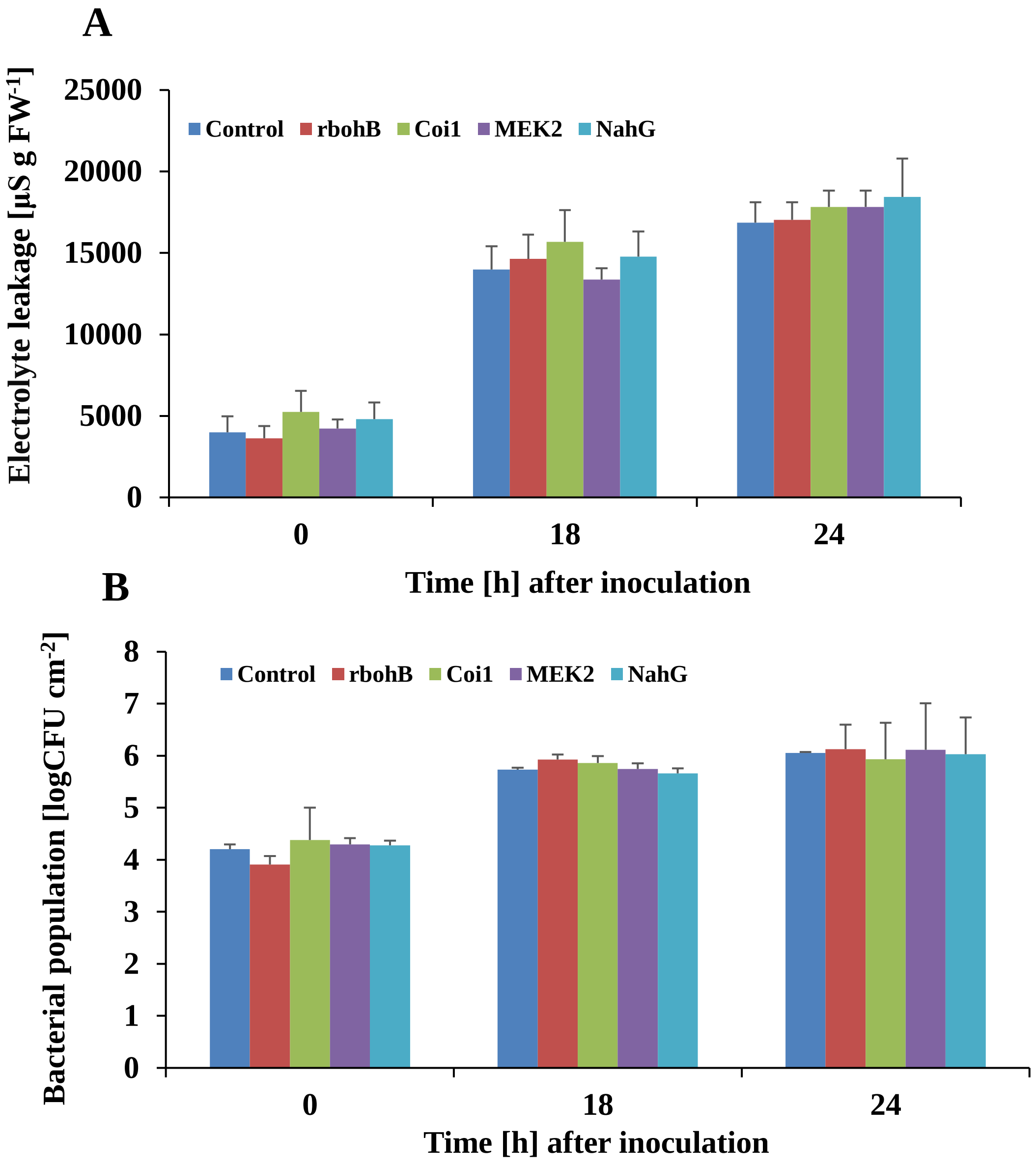

Figure S6. Bacterial population and hypersensitive cell death induction by incompatible *Ralstonia solanacearum* 8107 in *Nbcoi1*, *NbrbohB* and *NbMEK2*-silenced plants and NahG plant.

Control, *Ncoi1*-, *NbebohB* and *NbPLCNEK2*-silenced plant leaves were infiltrated with *R. solanacearum* strain 8107. (A) Cell death induced by *R. solanacearum* was determined in Control (gray bar), *NbPLC3-1*-silenced (VIGS; black bar) and *NbPLC3-2*-silenced (VIGS; white bar) plants by measuring the ion conductivity levels. Values represent means ± SDs (n = 5). (B) The bacterial populations of *R. solanacearum* 8107 in Control (gray bar), *NbPLC3-1*-silenced (VIGS; black bar) and *NbPLC3-2*-silenced (VIGS; white bar) plants were determined by plating at specified time points. Values are means ± SDs of five replicate experiments.

Table S1 Primers used in this paper

Primers	Sequence [5`-3`]	Purpose	Reference
PLC3-1S	ATTCTTTTATACGCGGAATAACCAC	Full length cDNA	This study
PLC3-1A	CGGCAGTGTTCAAATTGATTTG	Full length cDNA	This study
PLC3-2S	CAACAATCCATTATAATCCCAATTTTTATAC	Full length cDNA	This study
PLC3-2A	TATCATTTTGTTGATACTATAATTTGAACCTTG	Full length cDNA	This study
PLC3sVIGSS	GTCGACGATTCACCATGATATGAATGAG	VIGS vector	This study
PLC3sVIGSR	GTCGACGAATCGGGAGAAAACAGCATG	VIGS vector	This study
PLC3-1VIGSS	GTCGACTTGTTTTCTCTTATGTTTCTGTGTT	VIGS vector	This study
PLC3-1VIGSA	GTCGACTTGTCCACCTAAAGCTTAGATAAAT	VIGS vector	This study
PLC3-2VIGSS	GTCGACGTCGTTTGTTTTCTCTTATGTTC	VIGS vector	This study
PLC3-2VIGSA	GTCGACTATCATTTTGTTGATACTATAATTTG	VIGS vector	This study
RV20	CAGGAAACAGCTATGAC	Sequencing	Maimbo et al. 2007
M4	GTTTTCCCAGTCACGAC	Sequencing	Maimbo et al. 2007
NbPLC3-1rtpF	GTTCCCATTAACAGTTCCTGAG	qRT-PCR	This study
NbPLC3-1rtpR	GCGACTCACAGTTAGACAAATTCGAAG	qRT-PCR	This study
NbPLC3-2rtpF	GTTCCCATTAACAGTTCCTGAA	qRT-PCR	This study
NbPLC3-2rtpR	TTTCGAATTTGTCTAACTGTGAGTCGT	qRT-PCR	This study
NbPR1rtpF	GGTCAACACGGCGAAAACC	qRT-PCR	Maimbo et al. 2007
NbPR1rtpR	GCCTTAGCAGCCGTCATGA	qRT-PCR	Maimbo et al. 2007
NbPR4rtpF	GGCCAAGATTCCTGTGGTAGAT	qRT-PCR	Maimbo et al. 2007
NbPR4rtpR	CACTGTTGTTTGAGTTCCTGTTCCT	qRT-PCR	Maimbo et al. 2007
NbICS1rtpF	ATTTCATGGTCCCTCA	qRT-PCR	Maimbo et al. 2007
NbICS1rtpR	TTCCTGGTCAAACAT	qRT-PCR	Maimbo et al. 2007
NbORPrtpF	AGGCACTAAGATTTCTC	qRT-PCR	Maimbo et al. 2007
NbOPRrtpR	GTTGATCCCATCTTTC	qRT-PCR	Maimbo et al. 2007
Nbhin1rtpF	CGGTCCACCCGAAGTCAA	qRT-PCR	Maimbo et al. 2007
Nbhin1rtpR	ACGCCGTCGATGAAGTAGTCA	qRT-PCR	Maimbo et al. 2007
NbUbe35rtpF	CTTCAGATTCGCACCGTTCT	qRT-PCR	Pombo et al. 2018
NbUbe35rtpR	CCAATGCTTCGCAATGTTCTC	qRT-PCR	Pombo et al. 2018
NbNQOrtpF	AAGGCGGTGGTCAAGAAA	qRT-PCR	Pombo et al. 2018
NbNQOrtpR	CAAACATACCAGCACCGAATG	qRT-PCR	Pombo et al. 2018
NbrbohBrtpF	AACAACCTCGGATACATTAT	qRT-PCR	Deang et al. 2015
NbrbohBrtpR	TGTAAATAGACCAGCCATAA	qRT-PCR	Deang et al. 2015
NbMEK2rtpF	GTGATTGGGCTAGTCTTAT	qRT-PCR	Deang et al. 2015
NbMEK2rtpR	AGTGGCTGGTCTATTCTGG	qRT-PCR	Deang et al. 2015
Nbcoi1rtpF	GCTCCACGCGATTACCAACGG	qRT-PCR	Song et al. 2020
Nbcoi1rtpR	CTGCCACCATCTCTTGCACACC	qRT-PCR	Song et al. 2020

Table S2 List of plasmids used in this study.

Plasmid	Relevant characteristics	Reference
pMD20	Cloning vector pMD20, Amp^r	Takara Bio
pMD-NbPLC3-1 full	pMD20 carrying full length sequence of *NbPLC3-1* cDNA	This study
pMD-NbPLC3-2 full	pMD20 carrying full length sequence of *NbPLC3-2* cDNA	This study
pMD-NbPLC3s	pMD20 carrying identical sequence of *NbPLC3-1 and NbPLC3-2* fragment for VIGS	This study
pMD-NbPLC3-1	pMD20 carrying *NbPLC3-1* fragment for VIGS	This study
pMD-NbPLC3-2	pMD20 carrying full length *NbPLC3-2* fragment for VIGS	This study
pPVX201	pPVX binary vector, Km^r	Baulcombe *et al.*, 1995
pPVXPLC3s	pPVX binary vector containing identical sequence of *NbPLC2-1* and *NbPLC3-2* fragment for VIGS	This study
pPVXPLC3-1	pPVX binary vector containing PLC3 -1 cDNA fragment for VIGS	This study
pPVXPLC3-2	pPVX binary vector containing PLC3 -2 cDNA fragment for VIGS	This study
P35S-AvrA	pMDC32-derived plasmid that contains the full-length *avrA*	Poueymiro et al. 2009
P35S-INF1	pGR106 binary vector carrying *PR1a:Inf1* fusion gene	Huitema *et al.*, 2005
P35S-MEK^{DD}	pGreen binary vector carrying constitutive active form of MEK	Katou et al. 2003
P35S-TMGMV-CP	pRI101-AN vector carrying coat protein gene from TMGMV	Gupta et al. 2013
P35S-L1	pRI101-AN vector carrying *L1* gene from papper	Gupta et al. 2013
